# The TransEuro open-label trial of human fetal ventral mesencephalic transplantation in patients with moderate Parkinson’s disease

**DOI:** 10.1038/s41587-025-02567-2

**Published:** 2025-05-02

**Authors:** Roger A. Barker, Nicholas P. Lao-Kaim, Natalie Valle Guzman, Dilan Athauda, Hjalmar Bjartmarz, Anders Björklund, Alistair Church, Emma Cutting, Danielle Daft, Viswas Dayal, Stephen Dunnett, Amy Evans, Shane Grealish, Naomi Hannaway, Xiaoling He, Sam Hewitt, Zinovia Kefalopoulou, Philipp Mahlknecht, Antonio Martín-Bastida, Krista Farrell, Sarah Moore, Harry Bulstrode, Tagore Nakornchai, Jenny Nelander-Wahlestedt, Linnea Roupé, Gesine Paul, Kathryn Peall, Anne Rosser, Adriana Roca-Fernández, Sophie Rowlands, Anne-Marie McGorrian, Caroline Scherf, Ngoc Nga Vinh, Victoria Roberton, Claire Kelly, Mariah Lelos, Eduardo Torres, Kate Shires, Rachel Hills, Debbie Williams, Andreas-Antonios Roussakis, Krista Sibley, Pamela Tyers, Ruwani Wijeyekoon, Caroline Williams-Gray, Thomas Foltynie, Paola Piccini, Robert Morris, Stanley E. Lazic, Olle Lindvall, Malin Parmar, Hakan Widner

**Affiliations:** 1 John van Geest Centre for Brain Repair, Department of Clinical Neurosciences and Cambridge Stem Cell Institute, University of Cambridge, Cambridge, UK; 2 Department of Brain Sciences, Imperial College London, Hammersmith Hospital, London, UK; 3 Department of Clinical & Movement Neurosciences, UCL Institute of Neurology, London, UK; 4 Department of Neurosurgery, Skånes University Hospital and Lund University, Lund, Sweden; 5 Department of Neurology, Skånes University Hospital and Lund University, Lund, Sweden; 6 Division of Psychological Medicine and Clinical Neurosciences, Cardiff University, Cardiff, UK; 7 Lund Stem Cell Center and Division of Neurology, Department of Clinical Sciences Lund, Lund University, Lund, Sweden

**Keywords:** Ageing, Physiology

## Abstract

Transplantation of human fetal ventral mesencephalic tissue in individuals with Parkinson’s disease has yielded clinical benefits but also side effects, such as graft-induced dyskinesias. The open-label TransEuro trial (NCT01898390) was designed to determine whether this approach could be further developed into a clinically useful treatment. Owing to poor availability of human fetal ventral mesencephalic tissue, only 11 individuals were grafted at two centers using the same tissue preparation protocol but different implantation devices. No overall clinical effect was seen for the primary endpoint 3 years after grafting. No major graft-induced dyskinesias were seen, but we observed differences in outcome related to transplant device and/or site. Mean dopamine uptake improved at 18 months in seven individuals according to [^18^F]fluorodopa positron emission tomography imaging but was restored to near-normal levels in only one individual. Our findings highlight the need for a stem cell source of dopamine neurons for potential Parkinson’s disease cell therapy and provide critical insights into how such clinical studies should be approached.

## Abstract (web summary)

Patients with Parkinson’s disease treated with fetal tissue are assessed 3 years after surgery.

## Main

Parkinson’s disease (PD) is a common neurodegenerative disorder that has been the subject of many different cell-based therapies predicated on the grounds that replacing dopamine cells or restoring the nigrostriatal dopaminergic pathway would be of therapeutic benefit. This is based on the fact that the loss of this pathway lies at the heart of the pathology. Although the treatment of PD with dopaminergic medications works very well, at least in the early stage of disease^1^, with time, these drugs result in side effects due to the nonphysiological stimulation of the dopaminergic receptors in the striatum as well as off-target effects, causing a range of autonomic and neuropsychiatric problems^2^. Thus, there has been much interest in replacing the lost A9 dopaminergic nigral neurons in PD through transplants of similar cells, most notably the developing dopaminergic neural precursor cells derived from the human fetal ventral mesencephalon (hfVM) collected after planned termination of pregnancies.

Previous clinical trials using allografted hfVM tissue to treat PD have had variable results. In the initial open-label studies, this approach worked well, with some individuals showing long-term clinical benefits that were associated with normalization of dopamine on positron emission tomography (PET) imaging and evidence of long-term graft survival and innervation of the host striatum^3^. However, subsequent double-blind placebo-controlled trials failed to show benefits and reported side effects such as disabling graft-induced dyskinesias (GIDs), which in some cases were so severe that deep brain stimulation was required. This, coupled to one case where normalization on dopamine PET imaging was seen without clinical benefit^4^ and the presence of α-synuclein Lewy body pathology in a fraction of grafted cells a decade after transplantation^5^, led many to conclude that hfVM tissue transplantation was not competitive as a therapy for PD^6^.

However, given that some individuals had shown remarkable long-lasting improvements in their PD motor features^7^ and considering the compelling logic of dopaminergic neuron replacement, we decided to test the clinical feasibility of an optimized hfVM tissue transplantation approach, taking into account factors that could influence the outcome, in a new open-label trial funded by the European Union in 2010 called TransEuro. The main aim was to determine whether intraputamenal transplantation of hfVM tissue could be further developed into a routine specialized therapeutic strategy for individuals with PD. We wanted to address the following specific scientific questions. (1) Is it possible for specialized centers to secure a sufficient supply of human fetal tissue to allow regularly scheduled clinical transplantations? (2) Can intraputamenal dopaminergic innervation be restored consistently to near-normal levels in individuals with PD using transplantation of hfVM? (3) Will the magnitude of restoration of putamenal dopaminergic innervation differ between centers (neurosurgical techniques used)? (4) Can major clinical improvements be seen consistently in grafted patients when intraputamenal dopaminergic innervation is restored to near-normal levels? (5) Can development of GIDs be avoided by selecting individuals with minimal l-DOPA-induced dyskinesias before the procedure and by using a more discrete dissection of the hfVM to avoid the inclusion of serotonergic neurons, which previously has been linked to the development of GIDs^8^?

Here, we report the predefined 3-year outcome of the TransEuro trial. Grafting took place between 2015 and 2018 and involved 11 individuals at two surgical sites: Cambridge, United Kingdom (*n* = 8 individuals), and Lund, Sweden (*n* = 3 individuals). Of these individuals, ten received sequential bilateral grafts of hfVM tissue obtained from three fetuses per side of the brain. One individual had a unilateral graft and did not wish to proceed to a second graft because of the unpredictability of surgical planning and the associated psychological stress. All individuals were followed for 3 years, having received 12 months of triple immunosuppression after the second transplant (or first transplant in the one case of unilateral grafting). The primary outcome measure was their Unified Parkinson’s Disease Rating Scale (UPDRS) Part III motor ‘OFF’ score. We also followed a separate group of 16 individuals from our ongoing TransEuro natural history study (see Barker et al.^9^) who did not undergo any surgery but underwent the same clinical assessments and PET imaging. This group served as a control group against which to compare our grafted patient cohort. Predefined secondary measures were examined along with PET imaging that investigated dopaminergic and serotonergic markers.

Overall, in this small group of patients, we found no obvious benefit from the hfVM transplants across the grafted cohort, comparing against their baseline values and against the nongrafted natural history control group. However, evidence of a positive correlation between clinical effect and number of surviving hfVM-derived dopamine cells, as evident in changes in dopaminergic PET imaging, was seen in some individuals. In addition, there were differences in outcomes between the surgical sites, which may relate to the device used to deliver the cells given that the tissue preparation was standardized between centers. Finally, changes were seen after grafting on [^11^C]3-amino-4-(2-dimethylaminomethylphenylsulfanyl)-benzonitrile ([^11^C]DASB) PET imaging, and in three patients nondisabling GIDs were observed.

## Results

### Baseline patient demographics and outcomes analyzed

The basic demographics of the transplant and control patient cohorts are presented in Table 1. Thirty-six individuals were randomized to join the transplant arm of the study, but nine withdrew during the prerandomization assessment. Thus, 27 patients were recruited, of which 11 ended up being grafted, and 16 remained as nongrafted PET ‘control’ individuals. No patients were excluded because of significant ventral striatal 6-[^18^F]fluoro-l-3,4-dihydroxyphenylalanine ([^18^F]FDOPA) loss, and all 11 patients approached for grafting after baseline imaging consented and were enrolled, with the exception of 1 who did not wish to be treated with immunosuppressive drugs.

**Table 1:** Demographic data of the transplant population and nongrafted control individuals who were assessed using identical protocols including PET imaging

	Total transplant	Total control	Sweden transplant	Sweden control	UK transplant	UK control
***N***	11	16	3	7	8	9
**Age (years)**	51.8 (9.2)	54.6 (4.7)	43.6 (0.25)	52.3 (3.9)	54.9 (9.03)	56.4 (4.6)
**Men/women**	9M/2W	14M/2W	2M/1W	7M/0W	7M/1W	7M/2W
**UPDRS III OFF**	31 (9.2)	23 (4.7)	28 (21.0)	25 (11.0)	32 (6.0)	22 (8.5)
**UPDRS III ON**	20 (10.9)	18 (8.7)	23 (19.7)	20 (11.5)	19 (7.5)	16 (6.0)
**UPDRS IV OFF time (min)**	30 (44)	30 (44)	30 (44)	120 (178)	45 (67)	0 (0)
**UPDRS IV ON time (min)**	900 (205)	924 (258)	930 (131)	797 (360)	889 (233)	1,023 (53)
**Hauser Diary OFF time (min)**	163 (209)	140 (246)	193 (283)	224 (322)	150 (196)	56 (100)
**Hauser Diary ON time (min)**	862 (186)	872 (280)	863 (289)	794 (372)	861 (154)	950 (130)
**l-DOPA equivalent daily dose (mg)**	516.5 (360.1)	490.3 (352.2)	426.4 (107.3)	658.5 (401.6)	550.3 (420.9)	359.4 (260.0)

Values are shown as means (s.d.), except for *N*, number of men (M) and women (W) and UPDRS IV OFF, which shows the median and median absolute deviation due to a skewed distribution.

The outcomes evaluated were as follows:

- Primary outcomes
  ° Change in motor UPDRS (UPDRS Part III) in defined OFF state at 36 months after last transplantation (OFF being defined as receiving no dopamine therapy for 12 h before the assessment or longer in the case of long-acting dopamine agonists (for example, ropinirole slow release))
- Secondary outcomes
  ° Change in timed motor tasks at 36 months after transplantation
  ° Dyskinesias present, both OFF and ON, at 36 months after transplant
  ° Change in l-DOPA equivalent daily dose at 36 months after transplantation
  ° Number of patients on l-DOPA therapy at 36 months after transplantation
  ° Change in patient-reported OFF time at 36 months after transplantation
  ° Change in quality of life outcome measures as reported by the patient (Parkinson’s Disease Questionnaire-39 (PDQ-39))
  ° Changes in [^18^F]FDOPA PET in transplanted patients 36 months after transplantation

### Primary outcome

**Fig. 1:**
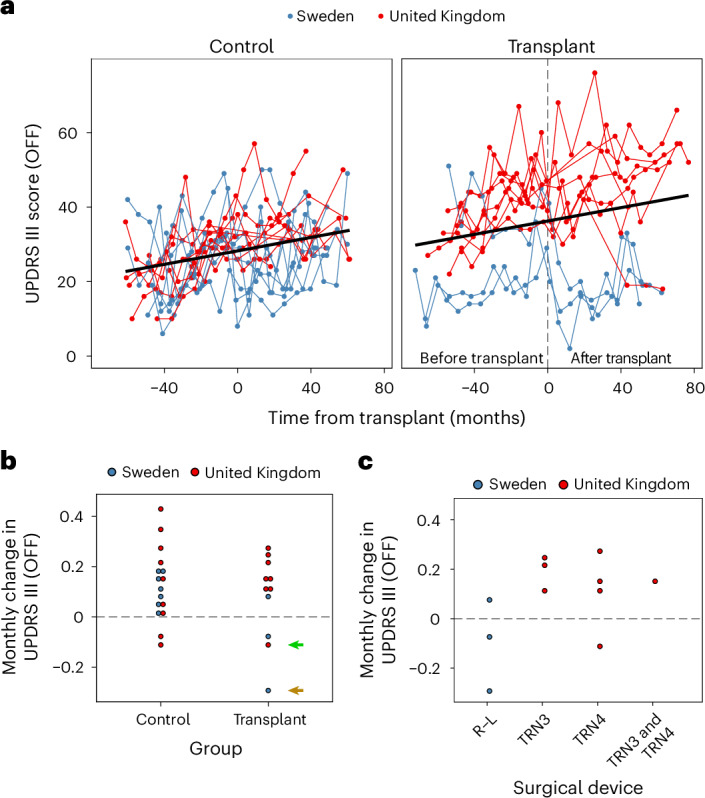
Change in UPDRS Part III in the defined OFF period. **a**, Individual trajectories. Time since entering the study was mean centered for the control individuals to align the *x* axes with the transplanted group. **b**,**c**, Average change per month by group (**b**) and device used (**c**). The green arrow indicates patient 79, and the brown arrow corresponds to patient 60 with near normalization on their dopamine PET scans.

**Fig. 2:**
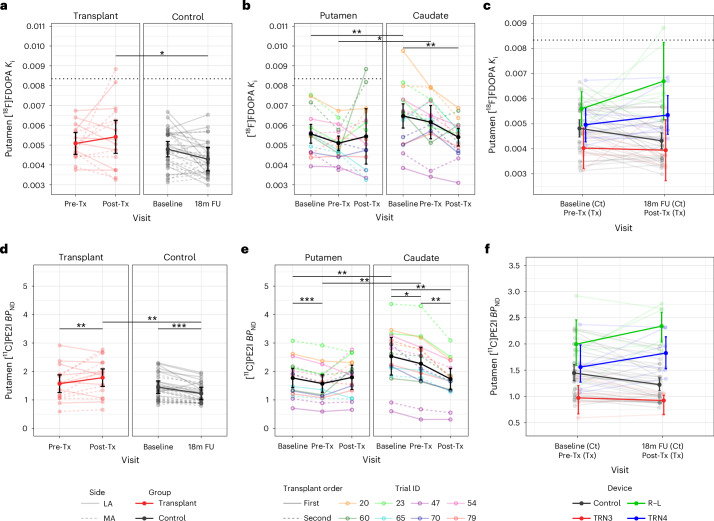
Analyses for dopaminergic PET imaging. Results of mixed two-way analyses of covariance (ANCOVAs) for bilateral putamen [^18^F]FDOPA *K*_i_ (**a**; *P* = 0.066) and [^11^C]PE2I *BP*_ND_ (**d**; *P* = 0.000062) including group (transplant: *n* = 8; control: *n* = 16) and visit (before transplant (Pre-Tx)/baseline and after transplant (Post-Tx)/18-month follow-up (18m FU) for transplant/control groups, respectively) as independent variables, adjusting for mean-centered age at baseline. Results of two-way repeated measures ANCOVAs within the transplant group (*n* = 8) are also shown for [^18^F]FDOPA *K*_i_ (**b**; *P* = 0.025) and [^11^C]PE2I *BP*_ND_ (**e**; *P* = 0.000057). Strip plots illustrate two-way interactions between region (bilateral putamen and caudate) and visit (baseline, before transplant and after transplant), adjusting for mean-centered age and disease duration at baseline. Bootstrapped means and 95% confidence intervals (bias-corrected and accelerated procedure, 10,000 replicates) by surgical device (TRN3/TRN4/R–L/control) and visit (before transplant/baseline and after transplant/18-month follow-up for transplant/control, respectively) were generated considering data from all putamen unilaterally and plotted for both [^18^F]FDOPA *K*_i_ (**c**; device *n* = 5 (TRN3), 8 (TRN4), 6 (R–L), and 32 (control)) and [^11^C]PE2I *BP*_ND_ (**f**; device *n* = 4 (TRN3), 6 (TRN4), 6 (R–L) and 32 (control)). Partially transparent lines represent data from individuals, whereas opaque lines and error bars represent estimated marginal means and 95% confidence intervals, respectively. Dotted horizontal lines for putamenal [^18^F]FDOPA *K*_i_ (**a**–**c**) represent the lower bound (pooled mean – 2 s.d.) for a healthy older cohort (*n* = 6 studies; total *n* = 71). Significance was analyzed by Tukey-adjusted post hoc tests; *P < 0.05; **P < 0.01; ***P < 0.001 (two sided); Ct, control; MA, most affected side; LA, least affected side.

The primary outcome was defined as the OFF UPDRS Part III score 36 months after the last transplant. This showed no obvious difference according to randomized transplant and control allocation compared to patient baseline scores. The primary outcome measure is further described in Fig. 1a, which plots the patient trajectories by group and site. Figure 1b plots the monthly UPDRS Part III (OFF) change by group, and Fig. 1c plots only the transplant group over time by site. Given that we used different devices at the two surgical sites, while using the same source and protocol for preparing the tissue (including standardized landmarks for dissection, as illustrated in Supplementary Fig. 1) in addition to standard operating practices for tissue preparation, we also undertook an exploratory analysis of changes in this measure by surgery site. We found that there were differences between the two surgical sites, which can only be described qualitatively as there are too few patients to perform meaningful quantitative statistical analyses. The three patients grafted in Lund using the original Rehncrona–Legradi (R–L) device had a different clinical response than those grafted in the United Kingdom using two slightly modified newly constructed in-house copies of this device (TRN3 and TRN4; Fig. 1c). Namely, these patients had more of a positive benefit from the transplant than the patients grafted in the United Kingdom. There could be other explanations for these differences, such as different neurosurgeons completing the procedure, the patients in Lund being less advanced at the time of surgery and differences in the cellular composition of the graft preparations due to the use of donors of different gestational age (see below and Fig. 2).

### Secondary outcomes

A large number of secondary measures were examined. The largest changes were seen in the l-DOPA equivalent daily dose and the percentage of time patients reported being in the OFF state (Table 2). For both measures, patients who were transplanted had better outcomes than control individuals, namely lower l-DOPA equivalent daily dose and reduced time in the OFF state. We report specifically on changes in the equivalent doses of l-DOPA medication taken at the end of the trial compared to just before their first transplant to determine whether graft implantation lowered their requirements for such medication.

**Table 2:** Secondary outcomes in the transplanted versus control patient cohort

Secondary outcome	Control	Transplant
Timed motor tasks, OFF (min)	−0.84 (11.8)	1.41 (16.3)
Timed motor tasks, ON (min)	3.87 (12.8)	−8.41 (20.7)
Dyskinesias, OFF (present/not present)	0/12^a^	3/8
Dyskinesias, ON (present/not present)	6/10	5/6
l-DOPA equivalent daily dose (mg)	306 (332)	15.6 (237)
Number of patients on l-DOPA therapy	15/0	11/0
Patient % reported OFF time	4.23 (12.0)	−7.75 (13.1)
Quality of life (PDQ-39)	10.1 (11.8)	3.6 (5.3)
[^18^F]FDOPA PET	Planned 36-month scanning could not be performed	

Values represent mean (s.d.) change from baseline to 36 months or counts (no/yes) at 36 months for dyskinesias with l-DOPA therapy (ON/OFF).

^a^Some of the control individuals did not want to come off medication, which is why this number is 12 and not 16.

### PET imaging findings

Imaging analysis was restricted to those patients who had received bilateral transplants and completed the imaging protocol at the 18-month postsurgical time point (*n* = 8) and control patients who had completed two [^18^F]FDOPA scans (*n* = 16). The complete set of dopaminergic PET scans for these patients who were grafted is shown in Supplementary Fig. 4. There was a trend toward a significant group by visit interaction (*F*_1,21_ = 3.76, *P* = 0.066), in which a significantly higher mean putamenal [^18^F]FDOPA *K*_i_ in the transplant group was seen at the post-transplant visit than in the control group at the 18-month follow-up (Fig. 2a). We dropped disease duration from the model because of assumption violation, although its inclusion produced similar results. Further analysis of the [^18^F]FDOPA scans in the transplant group revealed a significant region by visit interaction (*F*_2,10_ = 5.47, *P* = 0.025). Although the caudate continued to show loss of signal over time, this was reduced in the putamen with some stabilization of signal after transplantation (Fig. 2b).

The patterns were largely similar for [^11^C]PE2I; putamenal binding continued to decrease as expected for the control group but significantly increased following transplant in the grafted group (*F*_1,21_ = 24.84, *P* < 0.001; Fig. 2d). Disease duration was removed as it did not satisfy model assumptions, although results were similar when included. Within the transplant group, there was a significant region by visit interaction (*F*_2,10_ = 30.26, *P* < 0.001), whereby continued decreases in [^11^C]PE2I nondisplaceable binding potential (*BP*_ND_) were observed for the caudate but remained stable in the putamen (Fig. 2e).

Although not included in the models, bootstrapped means and 95% confidence intervals suggested that the most affected side of the putamen ([^18^F]FDOPA *K*_i_: 0.0047 (0.0044, 0.0051) to 0.0055 (0.0043, 0.0068); [^11^C]PE2I *BP*_ND_: 1.30 (1.010, 1.59) to 1.79 (1.260, 2.29)) responded better to surgery than did the least affected side ([^18^F]FDOPA *K*_i_: 0.0055 (0.0048, 0.0060) to 0.0054 (0.0046, 0.0066); [^11^C]PE2I *BP*_ND_: 1.86 (1.47, 2.29) to 1.79 (1.38, 2.15)). By contrast, the responses of the putamen transplanted first ([^18^F]FDOPA *K*_i_: 0.0050 (0.0045, 0.0058) to 0.0054 (0.0044, 0.0068); [^11^C]PE2I *BP*_ND_: 1.48 (1.10, 1.91) to 1.68 (1.24, 2.10)) compared to those of the putamen transplanted second ([^18^F]FDOPA *K*_i_: 0.0052 (0.0046, 0.0057) to 0.0055 (0.0045, 0.0065); [^11^C]PE2I *BP*_ND_: 1.67 (1.35, 2.16) to 1.90 (1.42, 2.32)) were similar.

We did not recruit healthy volunteers in this trial; however, across published [^18^F]FDOPA studies in healthy older individuals using similar analysis methods^10–15^, we can estimate a pooled mean *K*_i_ ± s.d. of 0.0107 ± 0.00119. Thus, [^18^F]FDOPA *K*_i_ in the transplant group increased from 47.6% to 50.8% of the normative level, whereas that in the control group decreased from 44.7% to 39.9%. Only patient 60 exhibited [^18^F]FDOPA *K*_i_ values within normal limits (2 s.d.) after transplant (Figs. 2b and 4), and this patient showed the largest improvement in their clinical scores.

**Fig. 3:**
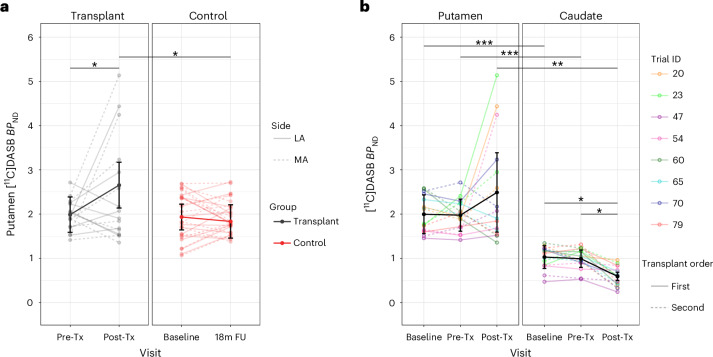
Analyses for 5-HT [^11^C]DASB PET imaging. **a**, Results of the mixed two-way ANCOVA for the bilateral putamen including group (*n* = 8 (transplant) and 14 (control)) and visit (before transplant/baseline and after transplant/18-month follow-up for transplant/control groups, respectively) as independent variables (*P* = 0.031). **b**, Within the transplant group (*n* = 8), a two-way repeated measures ANCOVA revealed an interaction between region (bilateral putamen and caudate) and visit (baseline, before transplant and after transplant; *P* = 0.038). Both analyses were adjusted for mean-centered age and disease duration at baseline. Partially transparent lines represent data from individuals, whereas opaque lines and error bars represent estimated marginal means and 95% confidence intervals, respectively. Significance was assessed by Tukey-adjusted post hoc tests; *P < 0.05, **P < 0.01 and ***P < 0.001 (two sided).

**Fig. 4:**
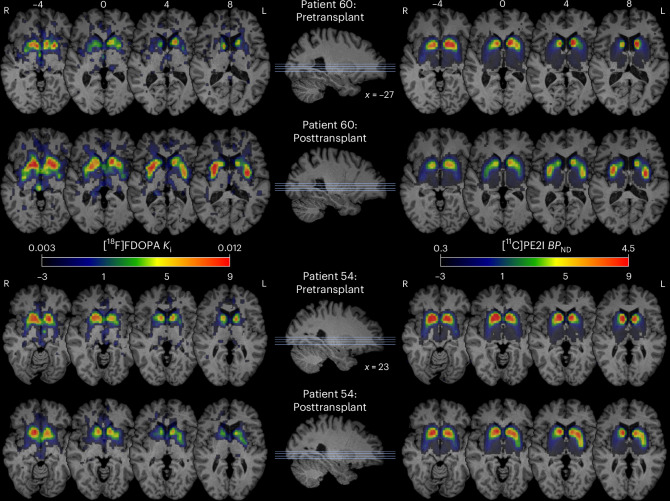
Dopaminergic PET after hfVM transplantation. Representative [^18^F]FDOPA (left) and [^11^C]PE2I (right) parametric images for two bilaterally transplanted patients whose postoperative levels of dopamine synthesis and transporter expression were either high (patient 60) or comparatively modest (patient 54). Dopaminergic PET data are overlaid onto patient T1-weighted magnetization prepared rapid gradient echo (MPRAGE) scans rigidly aligned to the Montreal Neurological Institute (MNI) template and displayed as axial slices covering the putamen; R, right; L, left.

Compared to the patients in our previous studies with fetal grafts (Lund series: patients 3–10 and 12–16; Supplementary Table 1), both the postoperative [^18^F]FDOPA *K*_i_ increases relative to normal and the percentage [^18^F]FDOPA *K*_i_ change relative to before the operation were substantially lower in the TransEuro group (3.22 versus 20.84% and 6.92 versus 70.43%, respectively). The amount of implanted tissue did not differ significantly between the groups. Similar to Lund patients 12–16, the tissue implanted in the TransEuro patients had been exposed to tirilazad mesylate to improve the survival of grafted dopaminergic neurons. Despite the amount of implanted tissue being similar in these groups, the postoperative [^18^F]FDOPA *K*_i_ increases relative to normal and the percentage [^18^F]FDOPA *K*_i_ change compared to before the operation were much higher in the previously grafted patients (17.28 and 62.83%, respectively).

[^11^C]DASB *BP*_ND_ analyses revealed a significant group by visit interaction (*F*_1,18_ = 5.47, *P* = 0.031), with putamenal serotonin (5-HT) innervation increasing after transplant in the grafted group compared to before transplant and control values (Fig. 3a). Within the transplant group, there was a significant region by visit interaction (*F*_1.05,5.27_ = 7.46, *P* = 0.038 (Greenhouse–Geisser corrected)) driven by caudate 5-HT innervation decreasing from before to after transplant while remaining stable in the putamen (Fig. 3b). These data were also evaluated after Box–Cox transformation given the mild leptokurtic residual distribution, but results were similar using the untransformed data.

In addition, with respect to dopamine imaging, bootstrapped mean and 95% confidence intervals were generated on the surgical device used by using the bias-corrected and accelerated procedure, 10,000 replicates and all available transplant cases in which postsurgical dopaminergic imaging was conducted (*n* = 10). For both [^18^F]FDOPA *K*_i_ and [^11^C]PE2I *BP*_ND_, greatest improvements appeared to occur with the R–L device, whereas TRN3 tended to confer the worst outcome (Fig. 2c,f).

Finally, changes in both putamenal dopaminergic parameters over the transplant period were found to be positively correlated with the Abnormal Involuntary Movement Scale (AIMS) total and global severity scores (*P* < 0.05). Responses to l-DOPA, calculated as the change in UPDRS Part III score following l-DOPA dosing, tended to improve with increasing [^11^C]DASB *BP*_ND_:[^18^F]FDOPA *K*_i_ ratio. No further significant correlations were found (Supplementary Table 2).

### Safety

All 11 of the patients in the transplant arm and 12 of the 16 (75%) patients in the control arm had one or more adverse events after transplantation (or postequivalent time point for control patients), with a total of 430 and 54 adverse events, respectively. The occurrence of adverse events is grouped according to their Medical Dictionary for Regulatory Activity System Organ class in Supplementary Table 3. Two hundred and sixty five of the 430 recorded adverse events (61.6%) in the transplant group related to a nonclinically significant abnormal investigation result. These mainly related to blood test results in the postsurgical setting, often likely related to immunosuppression. The transplant arm also underwent a much more intensive visit and investigative schedule than the control arm, particularly with regular postsurgery safety visits where blood tests were performed and adverse events were recorded. No abnormal investigation results were recorded for the control group as these patients did not have routine blood monitoring as part of their participation in the TransEuro trial.

As the neurological adverse events were much higher in the transplant group than in the control group, we also explored these in more detail. In the transplant group, there were 37 neurological adverse events among nine patients, of whom seven had at least 1 adverse event that was related to worsening parkinsonism. This tended to occur in the immediate postoperative procedure, as is typically seen in patients with PD undergoing major surgery with a general anesthetic. Three patients developed what seemed to be dyskinesia in the OFF state, which are assumed to be GIDs; in all cases they were mild and not disabling. Six patients who were grafted had at least one other neurological adverse event (there were two intracerebral hemorrhages that resulted in no neurological sequelae). By contrast, there were only four neurological adverse events documented in the control group, two of which were related to PD (increased OFF period time and worsening freezing).

Five of the 11 (45.5%) patients in the transplant arm and 3 of the 16 (18.8%) patients in the control arm had serious adverse events, totaling seven and five serious adverse events, respectively. There were no deaths among either the control or transplant participants of the study.

Five of the seven serious adverse events in the transplant group were deemed to be related to the transplant procedure or immunosuppression (Supplementary Table 4). Three were procedure related (two intracerebral hemorrhages and one wound dehiscence), and two were related to immunosuppression (azathioprine-induced colitis requiring hospital admission and a Kaposi’s sarcoma, both of which resolved on modifying the immunosuppression). The intracerebral hemorrhages were detected 2 days after surgery on routine scans. One (in a patient undergoing surgery in Lund), which was located in the immediate vicinity of the burr hole and thought to relate to diathermy of bridging veins, caused minor symptoms that resolved within 2 weeks. In the other case, in a patient grafted in Cambridge, the hemorrhage was intraparenchymal and above the transplant site but was asymptomatic. Intracerebral hemorrhage has a 1% risk with any intracerebral procedure, and if we assume an independent 1% risk of hemorrhage per needle pass, then the occurrence of two hemorrhages in 105 needle passes in this study is not that unexpected. None of these severe adverse events resulted in long-term disability, although one did result in a transplant patient having to discontinue all immunosuppression before the planned 12-month time period.

One patient (patient 79) had both their transplants placed outside the target site, with both grafts placed laterally to the striatum. The major targeting error in this patient was in the *y* axis rather than the *z* axis, which would have been the case if there was a depth miscalculation relating to the device. There was also no calibration error in the stereotactic frame used. The trial was temporarily suspended when this was seen on the day 2 postoperative magnetic resonance imaging (MRI) scan following the first transplant. Neurosurgeons working on this study, and another from a separate independent neurosurgical center, met to discuss the case and look at all the relevant information and imaging. No obvious explanation could be given for this, and the same graft misplacement happened when the patient was grafted on the contralateral side. This misplacement of grafts was incorporated into the data analysis and highlighted in the plotted data and was not seen in any other patient.

## Discussion

The use of dopamine cell-based therapies for treating PD has a long history, with the first transplants having been done in the 1980s (ref. ^3^). Over the ensuing 35 years, the results from such an intervention have varied markedly. Some patients were able to come off their anti-PD medication altogether, with restoration of striatal dopamine levels to within normal limits as evidenced by PET imaging and at postmortem^3,7,16^. By contrast, other patients have shown no benefit and even side effects such as GIDs despite, in one case, an apparent survival of >300,000 transplanted dopamine neurons^4^. This failure to show any benefit was most obviously seen in two National Institutes of Health (NIH)-funded double-blind, placebo-controlled trials reported in 2001 and 2003 (refs. ^17,18^). The data from these clinical trials had been subjected to a limited meta-analysis, which highlighted factors that seemed to associate with an increased likelihood of a positive clinical outcome^16^. This analysis formed the basis for the design of the TransEuro trial. Our evidence from the current trial clearly indicates that, despite attempts to optimize our approach, transplantation of hfVM tissue is not a clinically feasible therapeutic strategy for patients with PD.

First, we show that despite major efforts from two collaborating centers, access to hfVM tissue is insufficient to allow for scheduling clinical transplantations. Thus, many planned surgeries in the TransEuro trial had to be canceled. In addition, our use of a simple randomization approach for patient allocation to the transplant versus control arms may have led to problems in balancing the two groups, which negatively affected data analysis.

Second, despite standardized dissections and tissue preparations, variation in the gestational age of the donors for each patient, the shortage of tissue available for each patient and the limited possibilities for standardization and quality control inherently lead to variation in outcome. Most notably, only one of eight bilaterally grafted PET-imaged patients showed near-normal levels of putamenal dopaminergic innervation, as evidenced by [^18^F]FDOPA PET imaging. This low efficacy in restoring putamenal dopaminergic signaling, which forms the mechanistic basis for a potential motor improvement, is unacceptable in the clinical setting. Consequently, the trial did not reveal any significant clinical benefit in the group of grafted patients compared to controls.

Third, we observed differences in the degree of restoration of putamenal dopamine levels between the two centers, which used different surgical devices but the same amount of implanted tissue (three hfVMs per putamen). This illustrates that surgical and device-related issues can significantly influence the outcome after transplantation of hfVM tissue, although exactly how is poorly understood.

Fourth, we were not able to avoid the development of GIDs, which occurred in a significant proportion of grafted patients (27%) in association with increased putamenal 5-HT innervation. This proportion of patients developing GIDs is not dissimilar to that reported in previous trials (15–54% in the two major NIH studies^17,18^), although in our study, all were mild and did not require additional neurosurgical interventions to treat them.

We did see some evidence that the implantation of hfVM tissue may have improved the clinical trajectory of treated PD when comparing pre- and postintervention scores in patients who received a graft and when comparing to a contemporary natural history control population of individuals with PD. This effect was modest, occurred in only seven individuals and slowly emerged over time, suggesting that it was unlikely to be a placebo effect. We sought to minimize investigator bias in the assessment of patients by comparing the similarity of scores using a blinded third rater who scored patient videos. These results, coupled to the imaging data on dopamine innervation at the site of grafting, further support the belief that the clinical response was due to surviving implanted dopaminergic neurons. This is also in line with the fact that both l-DOPA equivalent daily dose (LEDD) and reported OFF times were lower in grafted patients 36 months after surgery than in the natural history control individuals. We found no consistent major placebo effect associated with long-term follow-up (reinforced by a complete absence of clear clinical or imaging effects in a patient in which the tissue was not grafted into the correct target region).

This trial was originally designed in 2008 to consist of a small open-label study that would then inform a bigger double-blind, sham surgery-controlled trial^9^. hfVM tissue was chosen as the tissue to be implanted as no protocol existed at this time for the generation of authentic midbrain dopaminergic neurons from human pluripotent stem cells. The recent successes in the development of dopaminergic neurons from stem cells happened after the design and setup of our trial^9,19–23^. However, using hfVM tissue proved not to be possible because of insufficient tissue supply and the inability to provide fully standardized tissue preparations. Although care was taken to standardize the dissection and preparation of the tissue across different centers, there was a large variation in gestational age of donor fetuses available for each transplant. Additionally, the scarce amount of tissue available for each patient precluded any quality control and comparison of tissue preparations used for each patient^9^. Thus, we eventually ended up producing a smaller, open-label study at two sites. As a result, the interpretation of the data is limited given that only 21 transplants were performed in 11 patients over a 3-year period, and thus any conclusions we draw have to be seen as tentative and more anecdotal than definitive. This is especially the case given the limited long-term PET scanning we could complete because of restrictions related to the coronavirus disease 2019 (COVID-19) pandemic.

The variability of the results in our trial is similar to that seen in many other trials using this approach^16^ and will always be an issue when the final tissue preparation cannot be standardized and quality controlled, which fortunately has now become possible using stem cell-derived dopaminergic neuroblasts^19–23^. The use of stem cells also gives the possibility to exclude serotonergic neurons and their progenitors, which is not feasible using fetal tissue where these two cell populations reside in close proximity. However, other factors seemed to also contribute to the variability in our study, including the center where surgery was performed. This implicates factors such as patient selection, neurosurgical approach and tissue preparation, which we endeavored to standardize but we were ultimately unable to use the same device at the two surgical sites. The original aim to use the R–L device in both Lund and Cambridge was not possible as the device is not Conformité Européenne marked and thus could only be used in the hospital where it was made (Lund). An imitation device had to be manufactured at the Cambridge site based on the original R–L instrument, but it was clearly not identical and may have explained some of the between-site differences in outcome. In addition, the patients that were randomly selected for surgery out of the observational study at the two sites had slightly different baseline characteristics, with patients in Lund having slightly less advanced disease. This may account for the differences by site, namely younger, less advanced patients have better outcomes. However, the small numbers of patients prevent any formal analysis of this hypothesis. In our new ongoing STEM-PD trial, we now assess all patients jointly across centers before transplantation to ensure that baseline characteristics are more comparable.

Although overall the approach was well tolerated, it was not without complications with respect to surgical procedure and immunosuppression. There were two intracerebral hemorrhages (without any lasting neurological sequelae), three patients developed mild GIDs, and two patients developed complications with their immunosuppressive drugs that necessitated stopping some of the agents they were taking. This is a relatively high rate of complications, although ultimately none were disabling or caused major long-term problems for the patients. This level of complication may relate to the novelty of the approach and with time would likely lessen if the technique became more widely adopted, as was the case for deep brain stimulation for PD. Nevertheless, this will be an important area to monitor as the newer stem cell dopamine cell therapies are tested in trials. In this respect, stem cell therapies may have another advantage in that they will lack 5-HT contaminants. We sought to reduce the risk of GIDs by minimizing the level of 5-HT contamination within our grafted tissue using a restricted dissection approach, but this proved inadequate as evidenced by changes in 5-HT innervation after grafting.

Overall, the TransEuro trial has been highly informative for the development of dopamine cell-based therapies for PD even though it failed to deliver in terms of the original trial design and meet its primary outcome. Our results are especially pertinent as the field enters the first-in-human clinical trials of human pluripotent stem cells for PD. The present study did suggest that hfVM grafts can positively affect the natural history of treated PD in some patients, even if these effects were modest. The low clinical efficacy was, as indicated by the PET imaging data, likely due to low numbers of surviving dopaminergic neurons and poor reinnervation of the putamen in most patients. This conclusion is further supported by the imaging changes on PET when comparing the patients in our previous studies with hfVM grafts (Lund series: patients 3–10 and 12–16; Supplementary Table 1). Both the postoperative [^18^F]FDOPA *K*_i_ increases relative to normal and the percentage [^18^F]FDOPA *K*_i_ change relative to before the procedure were substantially lower in the TransEuro group (3.22 versus 20.84% and 6.92 versus 70.43%, respectively). Notably, the setup and execution of this trial has been instrumental in defining the structure of the new round of stem cell-based dopamine trials in PD that has now started, in which batch-manufactured, quality-controlled and standardized preparations of dopamine progenitors are transplanted^21,23^ (NCT04802733; ref. ^24^). In particular, long run-in periods of patients who are then grafted, the use of PET imaging to monitor dopamine cell survival and innervation density and contemporaneous natural history controls have all been shown to be valuable in assessing the safety and efficacy of such interventions and should be considered in all future stem cell-derived dopaminergic neuron therapy trials. In addition, ensuring that sufficient numbers of dopamine cells survive long term and innervate the transplant site, such that the dopamine innervation within the grafted striatum is restored to normal, will be critical. Relevant factors that we have identified here include the dose of cells implanted, the device used for delivery of the cell product and possibly age of the patients (that is, at an earlier disease stage). Thus, the TransEuro trial has provided insights not only in trial design but also in some of the critical factors that need to be taken into account in the new round of stem cell-based dopamine cell replacement trials in patients with PD.

## Methods

### Study design and participants

The TransEuro Transplant trial (NCT01898390) was a randomized, open-label study that recruited patients from five sites across the United Kingdom and Sweden (University of Cambridge, Cambridge, UK, Imperial College London, London, UK, the National Hospital for Neurology and Neurosurgery, London, UK, University of Cardiff, Cardiff, UK, and Skåne University Hospital, Lund, Sweden). Eligible participants were recruited from the ongoing observational TransEuro observational study^9^ and rescreened using the original observational study inclusion criteria with modifications (Supplementary Table 5) to ensure continued eligibility for transplantation. Thirty-six patients were recruited into the transplant trial and randomly allocated to the transplant arm of the study or the control arm. Six withdrew before any intervention, and three did not complete screening. Eleven patients, of the remaining 27, went on to receive hfVM transplants. Sixteen patients served as a control arm. These patients underwent the same PET and clinical examinations as the transplant arm but did not receive immunosuppression and did not undergo sham surgery.

### Ethical approval

Ethical permission was received for fetal tissue preparation and use at Cambridge (96/085), Cardiff (13/WA/0210ADD) and Lund (2013/432 and 2016/535). The transplant study was approved by the relevant ethical authorities in the United Kingdom and Sweden (REC reference number 10/H0304/77 in the United Kingdom and the Swedish Ethical Review Authority (Etikprövningsmyndigheten) in Lund (reference numbers 2011/290, 2014/877 and 2019-06529)).

### Changes in study design

The original study design was to transplant 20 patients in an open-label fashion, drawn from a larger natural history cohort of 150 participants, and the data obtained would then be used to calculate sample size needed for a larger double-blind placebo-controled trial. All of this was to be done over a 5-year period. However, 5 years into the natural history study, the first patient was grafted, and, at this point, a decision was made to stop the transplant trial when either all 20 patients had been grafted or 3 years had elapsed from the time of the first transplant. This decision was based on the following reasons:

1. it seemed unethical to prolong the study beyond this time as it was clearly showing that using this tissue source in a trial was not feasible in the United Kingdom and Sweden,
2. interpretation of the study would become extremely difficult if some patients had already reached their primary endpoint whereas others had still not been grafted, and
3. advances in human stem cell-derived dopamine cells meant that trials using this new source of more readily available cells were already entering the clinic^24^.

At the end of 3 years in 2018, a total of only 11 patients had been grafted (8 in Cambridge, UK, and 3 in Lund, Sweden). The reasons for this have been previously presented^9^. Thus, the time of the final data collection for our predefined primary endpoint for the last patient was in March 2021. Collection of the follow-up clinical and PET imaging data was delayed (and in some cases not possible) because of restrictions resulting from the COVID-19 pandemic that began in March 2020, and this included the 36-month PET imaging in the majority of patients. To try and standardize the timings to better align with the PET imaging analysis, we defined a pretransplant baseline as the visit immediately before the first transplant. We then elected to use the following as the key time points for the primary and secondary outcomes:

- 18 months: first visit at least 510 days after the last transplant surgery or after the baseline visit (control; 540 days = 30 × 18 months with 30 days leeway)
- 36 months: first visit at least 1,020 days after surgery (transplant) or after the baseline visit (control; 1,080 days = 30 × 36 months with 60 days leeway)

### Clinical assessments

Once randomized, participants continued trial visits as part of the observational TransEuro trial schedule every 6 months. During each visit, participants underwent a battery of clinical tests during an OFF state (with the OFF state being defined as the patient not having had any dopaminergic medications for 12 h before assessments or 24 h for long-acting dopamine agonists) and ON state (defined as at least 1 h after the patient had taken their regular morning dose medications). These assessments included UPDRS Part III, RUSH Dyskinesia Scale and AIMS. Patients also completed the UPDRS Parts I, II and IV, Addenbrookes’ Cognitive Examination-Revised and a series of other cognitive and PD-related assessments (Supplementary Table 6). Study data were collected and managed using REDCap electronic data capture tools hosted at the University of Cambridge.

### Imaging assessments

MRI and PET scanning were performed at Invicro, Hammersmith Hospital, London, UK. Patients had a structural MRI and [^11^C]PE2I, [^11^C]DASB and [^18^F]FDOPA PET scans at baseline and repeat scans just before surgery and at 18 months after their first transplant. The planned 36-month scanning could not be performed in sufficient numbers of patients because of the COVID-19 pandemic that began in 2020.

Image processing and kinetic modeling were conducted using MIAKAT v4.3.13 (Molecular Imaging and Kinetic Analysis Toolbox)^25^ implemented within MATLAB 2016b (Mathworks), SPM12 v7487 (Statistical Parametric Mapping, Wellcome Trust Centre for Neuroimaging) and FSL v6.0 (FMRIB Image Analysis Group)^26^.

Structural MPRAGE images were segmented and rigid registered to the MNI template, and for each patient, all visits were entered into serial longitudinal registration to create a midpoint average and associated deformation fields. The midpoint was used to define the putamen and caudate in accordance with previously published anatomy-based guidelines^27^. Cerebellar gray matter was isolated using DARTEL to estimate flow fields from the MNI template to native space, applying this to CIC Atlas v1.2 and masking with a gray matter segment. Dynamic PET images were motion corrected and co-registered with their corresponding MPRAGE, using the summed PET images as an intermediary and normalized mutual information as a cost function, in one interpolation step. Parcellations were then applied to the dynamic PET data to generate regional time–activity curves. For [^18^F]FDOPA, Patlak graphical analysis was used to quantify the uptake rate constant (*K*_i_), whereas for [^11^C]PE2I, Logan graphical analysis was used to quantify *BP*_ND_. For both, t* was set to 30 min. For [^11^C]DASB, *BP*_ND_ was estimated with Simplified Reference Tissue Model 2 (SRTM2). Cerebellar gray matter was used as a reference region for all tracers.

### Tissue preparation

Transplanted tissue was prepared from hfVM dissected from three fetuses collected after either medical or surgical abortions under full ethical approval. Tissue dissections were standardized across centers by established landmarks and documentation of each cut using photographs. The landmarks used for dissection are shown in Supplementary Fig. 1.

The collected tissue was stored for a maximum of 4 days in Hib(E) at 4 °C. On the day of surgery, three hfVMs were pooled and washed several times in DMEM (cGMP compliant, Life Technologies, A12861 01)/tirilazad mesylate (custom made to GMP grade, Rechon Life Sciences). The hfVMs were enzymatically digested in a mixture of Tryple E CTS (cGMP compliant, Life Technologies A12859-01) and Pulmozyme (Dornase-α, Roche) at 37 °C for 20 min. After incubation, the tissue was washed three to four times in dissociation medium DMEM/tirilazad mesylate/dornase-α to remove any Tryple E residue. The hfVMs were then dissociated very gently to produce a crude cell suspension, which was spun down and resuspended. Aliquots of 5 × 20 µl were prepared after confirmation of viability and transported to theater in a temperature-monitored cool box. Quality criteria to proceed with transplantation of hfVM cells was set at >80% cell viability on the day of implantation. Although insufficient tissue was a common problem given that the cell preparation had to be derived from at least three hfVMs per side grafted, only one cell preparation had a viability below that required for surgery. The crown rump length of the fetus varied between 15 mm (gestational age (weeks ± days) 7 + 6) and 35 mm (gestational age 10 + 2), and the final cell suspension viability was between 83 and 93%.

It is worth noting that in TransEuro, unlike previous hfVM transplant trials, the tissue was dissociated not using trypsin but using Pulmozyme, as the former could not be sourced at a clinical grade. Given the low number of patients transplanted, it could not be experimentally determined whether Pulmozyme was superior or not to trypsin and had impacted the final number of dopamine cells in the grafted tissue. In addition, we used tissue collected from medical terminations of pregnancy, not surgical terminations, as was the case in earlier trials^28^. This may also have had an impact on the final number of surviving dopaminergic cells within the graft, as might the time spent in hibernation media before the final tissue preparation and transplant surgery.

### Surgery

Neurosurgery was performed at one of two sites. All three Swedish patients underwent surgery at Skåne University Hospital, Lund, Sweden. All eight patients recruited at the UK sites had surgery performed at Cambridge University Hospital, Cambridge, UK. Each patient underwent two unilateral transplants within an interval of 1–5 months (3.88 ± 2.49 months) with imaging guidance for trajectory and stereotactic planning. Five trajectories were made per putamen using a transfrontal approach. Eight deposits of 2.5 μl were injected per trajectory for a total of 20 μl per trajectory. MRIs were performed after surgery to show the sites of tissue deposition (Supplementary Fig. 2), although only the needle tracts can be seen, not the transplant itself, as MRI cannot provide validated evidence for the integration of the grafted cells into the brain. Due to regulatory differences between countries, different surgical devices were used to deliver the transplants between the UK site and the Swedish site. The device used in Lund was the original R–L device used in previous open-label trials^28^, whereas in Cambridge, an in-house-manufactured version of this device was made (TRN3), which was subsequently modified (TRN4) part way through the trial. This modification was undertaken in response to feedback from the neurosurgeon using the device in Cambridge, and the needle in both devices had an internal diameter of 0.82 mm and an external diameter of 1.07 mm.

After surgery, patients were given prophylactic antibiotics and were started on a standard whole-organ immunosuppressant regimen of cyclosporin (titrated to serum levels of between 100 ng ml^–1^ and 200 ng ml^–1^), 2 mg per kg (body weight) per day azathioprine and 40 mg of prednisolone weaning to 5 mg over 12 weeks after a one-off dose of 1 g at the time of surgery. Immunotherapy was maintained for 12 months after the last transplant and was then stopped. During this time, patients also took all recommended prophylactic treatments for patients on this immunosuppressive regimen, namely co-trimoxazole three times a week, omeprazole, calcichew daily and alendronic acid once a week.

### Postsurgical visits

Patients were followed up 12, 24 and 48 h after surgery for routine postsurgical observations and blood tests. Two days after surgery, a postoperative brain MRI scan was performed to verify graft placement and examined for any perioperative hemorrhage. In addition to their regular study clinical assessment visits, patients also had safety visits at 7, 14, 21, 28 and 42 days and then 2, 3, 4, 5, 6, 9 and 12 months after surgery as well as blood testing to monitor their immunosuppression.

### Video rescoring

To maintain intersite and inter-rater reliability, all UPDRS Part III assessments were videotaped. A random selection (*n* = 25) of these corresponding to the key time points (pretransplant visit and 36-month post-transplant visit or equivalent for controls) were examined by an independent rater blinded to the patient’s transplant status and rescored. These videos did not reveal the patient’s surgical status as all patients were required to wear a hat at all assessments to hide the presence or absence of surgical scars so that their group allocation (transplant or control) could not be identified from the videos. These rescored UPDRS Part III scores were used in the evaluation instead of the original score; however, the overall concordance rate between the two UPDRS scores was high (Supplementary Fig. 3).

### Clinical outcomes

The primary outcome measure was defined as change in the UPDRS Part III score in the defined OFF state at 36 months after surgery compared to baseline. As the treatment is a dopamine therapy, and most likely to affect motor outcome, a motor score was felt to be the most appropriate measure, and the 36-month time point was chosen to allow sufficient time for any post-transplant benefits to evolve.

Secondary outcomes included a range of motor, nonmotor, quality of life and cognitive measures as well as changes in dopaminergic medication. These are summarized in the main text.

### Statistical analysis

Given the limitations of sample size, site to site variability and procedural and patient heterogeneity, it is debatable whether inferential analyses are relevant and interpretable. Therefore, no inferential statistics were used to assess the efficacy of the transplants on the primary or secondary outcomes.

Statistical analysis of the imaging data included all bilaterally transplanted patients who completed the multi-PET protocol at three time points (*n* = 8). We performed two-way mixed ANCOVAs with repeated measures to examine whether differences in mean putamenal [^18^F]FDOPA *K*_i_, [^11^C]PE2I *BP*_ND_ and [^11^C]DASB *BP*_ND_ values depended on group (transplant or control) and visit. Visit included pretransplant and 18 months post-transplant time points for the transplant group (*n* = 8) or baseline and 18-month follow-up time points for the control group, for which data were available for 16 patients for [^18^F]FDOPA *K*_i_ and [^11^C]PE2I *BP*_ND_ and for 14 patients for [^11^C]DASB *BP*_ND_. We also conducted a series of two-way repeated measures ANCOVAs to examine whether differences in mean striatal [^18^F]FDOPA *K*_i_, [^11^C]PE2I *BP*_ND_ and [^11^C]DASB *BP*_ND_ values depended on visit (baseline, pretransplant and post-transplant) and striatal region (putamen and caudate). The caudate was included as an internal control region given that it is also known to exhibit substantial dopaminergic neurodegeneration over time in PD. For both analyses, mean-centered age and disease duration at the first included time point were entered as continuous covariates where possible as they have been shown to be related to dopaminergic and serotonergic loss. Post hoc Tukey-adjusted pairwise comparisons of the estimated marginal means were conducted to evaluate pairwise differences where appropriate.

Spearman’s rank-order correlations were conducted to evaluate the relationship between the changes in PET parameters (posttransplant–pretransplant) and primary and secondary outcome scale change scores. For this purpose, observational data collected closest in time to the PET acquisitions were included for analysis.

Statistical analyses and visualizations were computed in R version 4.2.2 using the following packages: afex 1.3.0, car 3.1.2, emmeans 1.8.6, moments 0.14.1, geoR 1.9.2, rcompanion 2.4.30, rstatix 0.7.2, Hmisc 5.1.0, FSA 0.9.5, ggplot2 3.4.1 and ggpubr 0.6.0.

### Reporting summary

Further information on research design is available in the Nature Portfolio Reporting Summary linked to this article.

## Online content

Any methods, additional references, Nature Portfolio reporting summaries, source data, extended data, supplementary information, acknowledgements, peer review information; details of author contributions and competing interests; and statements of data and code availability are available at 10.1038/s41587-025-02567-2.

## Supplementary information

Supplementary Information Supplementary Tables 1–6, Figs. 1–4 and entire clinical trial protocol.

Reporting Summary

## Data Availability

Data that support the findings of this study are not openly available to protect study participants’ privacy. Data may be requested from the corresponding author upon reasonable request immediately and for a period of 36 months following article publication. Reasonable requests will be considered from researchers who provide a methodologically sound proposal. Data would be provided anonymized. Study documentation, including the protocol and statistical analysis plan, is available within the Supplementary Information.
