# Supplementary material for: The TransEuro open-label trial of human fetal ventral mesencephalic transplantation in patients with moderate Parkinson’s disease
Source: Nat Biotechnol. 2025 May 2;44(1):70–8. doi: 10.1038/s41587-025-02567-2 (PMC12807853; doi:10.1038/s41587-025-02567-2)
Supplement: Supplementary file 1 — Supplementary Tables 1–6, Figs. 1–4 and entire clinical trial protocol. [file 41587_2025_2567_MOESM1_ESM.pdf]

nature biotechnology

Supplementary information https://doi.org/10.1038/s41587-025-02567-2

The TransEuro open-label trial of human fetal ventral mesencephalic transplantation in patients with moderate Parkinson's disease

In the format provided by the authors and unedited

Supplementary Table 1

Comparison of PET transplant outcomes between the Lund series and current TransEuro trial. Lund series [^{18}F]FDOPA K_i estimates were derived for the transplanted putamina of patients 3-10 and 12-16 (Widner 1992, Wenning 1997, Hagell 1999, Brundin 2000), from scans immediately prior to transplant and up to 26 months post-surgery (Time). Only striatal (putamen + caudate) data were available for patients 5 and 6, but removing them from the analysis made negligible difference to the statistics. % calculations for TRANSEURO were conducted using an estimate of the healthy mean [^{18}F]FDOPA K_i, as described in text. Data presented are the mean ± SD of transplanted putamina/striata for TransEuro (n=16) and Lund series (n=25) patients, with the latter also sub-divided depending on VM tissue and patient treatment with (Pts 12-16, n=10) or without (Pts 3-10, n=15) tirilazad mesylate. Groups were compared using nonparametric Mann-Whitney U ($p^{M\text{-}W}$, two-sided) and Kruskal-Wallis ($p^{K\text{-}W}$) with post-hoc Bonferroni-Holm-adjusted Dunn tests (p^{D}, two-sided). 95% confidence intervals were constructed for Mann-Whintey and Kruskal-Wallis effect sizes by bootstrap resampling using the percentile method (10,000 replicates). VM = ventral mesencephalon, effect size *r* calculated as $Z/\sqrt{N}$, effect size η^2 calculated as $(H-k+1)/(n-k)$.

	**A. TransEuro**	**B. Lund Series**	***r* [95% CI], $p^{M\text{-}W}$** (A, B)		**C. Lund Series** Pts 3-10	**D. Lund Series** Pts 12-16	**η^2 [95% CI], $p^{K\text{-}W}$** (A, C, D)	**p^D** (A, C)	**p^D** (A, D)	**p^D** (C, D)
**Time (months)**	19.41 ± 5.30	17.46 ± 5.15	0.10 [0.00, 0.40], 0.530		17.57 ± 5.51	17.28 ± 4.85	-0.04 [-0.05, 0.18], 0.812	-	-	-
**Donor VMs**	3 ± 0	3.73 ± 1.18	0.14 [0.00, 0.46], 0.373		4.34 ± 1.12	2.81 ± 0.43	0.72 [0.45, 0.92], <0.001	0.002	0.012	<0.001
**[^{18}F]FDOPA K_i**										
**% of HV at Pre-Tx**	47.61 ± 7.99	31.58 ± 7.98	0.71 [0.54, 0.82], <0.001		32.30 ± 7.74	30.51 ± 8.62	0.49 [0.28, 0.69], <0.001	<0.001	<0.001	0.733
**% of HV at Post-Tx**	50.84 ± 15.47	52.42 ± 12.70	0.07 [0.00, 0.39], 0.659		55.52 ± 14.10	47.78 ± 9.01	0.01 [-0.05, 0.26], 0.311	-	-	-

**% change relative to HV**	+3.22 ± 13.55	+20.84 ± 10.28	0.62 [0.35, 0.81], <0.001		+23.21 ± 11.65	+17.28 ± 6.88	0.39 [0.11, 0.68], <0.001	<0.001	0.017	0.383
**% change relative to Pre-Tx**	+6.92 ± 29.96	+70.43 ± 39.89	0.70 [0.49, 0.83], <0.001		+75.49 ± 43.89	+62.83 ± 33.78	0.48 [0.22, 0.70], <0.001	<0.001	0.002	0.726

<u>REFERENCES:</u>

1. Widner H, Tetrud J, Rehncrona S, Snow B, Brundin P, Gustavii B, Björklund A, Lindvall O, Langston JW. Bilateral fetal mesencephalic grafting in two patients with parkinsonism induced by 1-methyl-4-phenyl-1,2,3,6-tetrahydropyridine (MPTP). N Engl J Med. 1992 Nov 26;327(22):1556-63. doi: 10.1056/NEJM199211263272203. PMID: 1435882
2. Wenning GK, Odin P, Morrish P, Rehncrona S, Widner H, Brundin P, Rothwell JC, Brown R, Gustavii B, Hagell P, Jahanshahi M, Sawle G, Björklund A, Brooks DJ, Marsden CD, Quinn NP, Lindvall O. Short- and long-term survival and function of unilateral intrastriatal dopaminergic grafts in Parkinson's disease. Ann Neurol. 1997 Jul;42(1):95-107. doi: 10.1002/ana.410420115. PMID: 9225690
3. Hagell P, Schrag A, Piccini P, Jahanshahi M, Brown R, Rehncrona S, Widner H, Brundin P, Rothwell JC, Odin P, Wenning GK, Morrish P, Gustavii B, Björklund A, Brooks DJ, Marsden CD, Quinn NP, Lindvall O. Sequential bilateral transplantation in Parkinson's disease: effects of the second graft. Brain. 1999 Jun;122 (Pt 6):1121-32. doi: 10.1093/brain/122.6.1121. PMID: 10356064
4. Brundin P, Pogarell O, Hagell P, Piccini P, Widner H, Schrag A, Kupsch A, Crabb L, Odin P, Gustavii B, Björklund A, Brooks DJ, Marsden CD, Oertel WH, Quinn NP, Rehncrona S, Lindvall O. Bilateral caudate and putamen grafts of embryonic mesencephalic tissue treated with lazaroids in Parkinson's disease. Brain. 2000 Jul;123 (Pt 7):1380-90. doi: 10.1093/brain/123.7.1380. PMID: 10869050

Supplementary Table 2: Correlation analysis between PET parameters (putamen) and clinical outcome measures. Data presented are Spearman correlation coefficients and associated two-sided *p*-values (n=8). *p < 0.05, [a]Calculated as UPDRS-III ON – OFF, [b]Sum of AIMS facial, oral, extremity and trunk items (1-7), [c]AIMS item 8, [d]Data unavailable for patient 79.

Change in Primary/Secondary Outcomes (Post-Tx – Pre-Tx)	[^{11}C]DASB BP_{ND}	[^{11}C]PE2I BP_{ND}	[^{18}F]FDOPA K_i	[^{11}C]DASB BP_{ND} / [^{18}F]FDOPA K_i
UPDRS-III				
Total (OFF)	0.263, 0.528	0.000, 1.000	-0.084, 0.844	0.024, 0.955
Total (ON)	-0.096, 0.821	-0.072, 0.866	-0.180, 0.670	-0.311, 0.453
Progression Rate (OFF)	0.095, 0.823	-0.524, 0.183	-0.429, 0.289	0.405, 0.320
Levodopa Responsiveness[a]	0.639, 0.088	-0.024, 0.955	0.157, 0.711	0.747, 0.033*
Time Motor Tasks				
Peg Board (OFF)	-0.036, 0.933	-0.707, 0.050	-0.395, 0.333	0.000, 1.000
Peg Board (ON)	0.143, 0.736	0.429, 0.289	0.452, 0.260	0.286, 0.493
Tap Test (OFF)	-0.036, 0.933	0.275, 0.509	0.144, 0.734	-0.252, 0.548
Tap Test (ON)	-0.548, 0.160	0.024, 0.955	0.214, 0.610	-0.548, 0.160
Sit, Stand, Walk (OFF)	-0.133, 0.754	0.241, 0.565	0.193, 0.647	-0.410, 0.313
Sit, Stand, Walk (ON)	-0.289, 0.487	0.024, 0.955	0.133, 0.754	-0.386, 0.346
AIMS				
Total[b] (OFF)	-0.027, 0.948	**0.825, 0.012***	0.646, 0.083	-0.261, 0.532
Total (ON)	0.098, 0.818	0.317, 0.444	0.122, 0.774	0.000, 1.000
Global Severity[c] (OFF)	0.055, 0.897	**0.784, 0.021***	**0.729, 0.040***	-0.179, 0.672
Global Severity (ON)	0.152, 0.719	0.355, 0.388	0.216, 0.608	-0.051, 0.905
LEDD	0.095, 0.823	-0.333, 0.420	-0.500, 0.207	0.071, 0.867
% Time.ON.with.Dyskinesia	0.393, 0.335	-0.063, 0.881	0.178, 0.674	0.304, 0.464

% Time OFF[d]	0.000, 1.000	-0.179, 0.702	0.000, 1.000	0.321, 0.482
PDQ39	0.429, 0.289	0.333, 0.420	0.286, 0.493	0.262, 0.531

Supplementary Table 3: Adverse Events by MedDRA System Organ Class/Preferred Term[1]

MedDRA System Organ Class<br>Preferred Term	Transplant		Control	
	Number of participants (%)[2]	Total number of aEs[3]	Number of participants (%)[1]	Total number of aEs[3]
**Blood and lymphatic system disorders**	**1 (9.1%)**	**1**	**0 (0%)**	**0**
Lymphopenia		1		0
**Cardiac disorders**	**2 (18.2%)**	**6**	**0 (0%)**	**0**
Sinus bradycardia		1		0
Supraventricular tachycardia		5		0
**Ear and labyrinth disorders**	**1 (9.1%)**	**1**	**0 (0%)**	**0**
Ear pain		1		0
**Endocrine disorders**	**1 (9.1%)**	**1**	**0 (0%)**	**0**
Hypothyroidism		1		0
**Eye disorders**	**3 (27.3%)**	**6**	**2 (12.5%)**	**5**
Cataract		0		1
Dry eye		1		0
Eye disorder		1		0
Eye haemorrhage		3		0
Eye pain		1		0
Glaucoma		0		1
Macular oedema		0		1
Vision blurred		0		2
**Gastrointestinal disorders**	**3 (27.3%)**	**5**	**1 (6.3%)**	**1**
Diarrhoea		1		0

Gastrooesophageal reflux disease		1		0
Nausea		1		0
Rectal haemorrhage		0		1
Toothache		2		0
**General disorders and administration site conditions**	**7 (63.6%)**	**17**	**2 (12.5%)**	**3**
Chest discomfort		2		0
Fatigue		2		0
Feeling abnormal		1		0
Generalised oedema		0		2
Hernia		0		1
Malaise		1		0
Oedema peripheral		6		0
Pain		3		0
Pyrexia		1		0
Swelling face		1		0
**Hepatobiliary disorders**	**2 (18.2%)**	**2**	**0 (0%)**	**0**
Cholelithiasis		1		0
Hepatic steatosis		1		0
**Immune system disorders**	**2 (18.2%)**	**2**	**0 (0%)**	**0**
Hypersensitivity		2		0
**Infections and infestations**	**10 (90.9%)**	**26**	**7 (43.8%)**	**8**
Candida infection		1		0
Cellulitis		1		0
COVID-19		1		1
Cytomegalovirus infection		3		0
Ear infection		1		0
Fungal skin infection		1		0

Gastroenteritis		0		1
Gingivitis		2		0
Herpes zoster		1		1
Hordeolum		0		1
Influenza		0		1
Lower respiratory tract infection		1		1
Nail infection		1		0
Nasopharyngitis		7		0
Pelvic abscess		0		1
Skin infection		1		0
Toxoplasmosis		0		1
Upper respiratory tract infection		1		1
Urinary tract infection		2		0
Vestibular neuronitis		1		0
Wound infection		1		0
**Injury, poisoning and procedural complications**	**6 (54.5%)**	**17**	**7 (43.8%)**	**11**
Bone contusion		0		1
Contusion		1		0
Craniofacial injury		0		2
Epicondylitis		2		0
Fall		5		3
Foot fracture		1		0
Fracture		0		1
Joint dislocation		0		1
Ligament injury		0		1
Meniscus injury		0		1
Nail injury		0		1
Post procedural oedema		1		0

Procedural complication		2		0
Upper limb fracture		1		0
Vascular access site bruising		3		0
Wound complication		1		0
**Investigations**	**11 (100%)**	**279**	**0 (0%)**	**0**
Activated partial thromboplastin time prolonged		1		0
Activated partial thromboplastin time shortened		2		0
Alanine aminotransferase decreased		2		0
Alanine aminotransferase increased		6		0
Blood albumin decreased		15		0
Blood albumin increased		1		0
Blood alkaline phosphatase abnormal		1		0
Blood alkaline phosphatase decreased		3		0
Blood alkaline phosphatase increased		1		0
Blood bilirubin increased		11		0
Blood creatinine abnormal		2		0
Blood creatinine increased		14		0
Blood glucose increased		2		0
Blood osmolarity decreased		1		0
Blood phosphorus decreased		2		0
Blood potassium abnormal		1		0
Blood potassium decreased		1		0
Blood potassium increased		3		0
Blood pressure decreased		1		0
Blood sodium decreased		9		0
Blood triglycerides increased		1		0
Blood urea abnormal		3		0
Blood urea increased		25		0

C-reactive protein increased		7		0
Eosinophil count decreased		2		0
Gamma-glutamyltransferase increased		1		0
Haematocrit abnormal		2		0
Haematocrit decreased		10		0
Haemoglobin decreased		30		0
Investigation abnormal		35		0
Lymphocyte count abnormal		8		0
Lymphocyte count decreased		9		0
Lymphocyte count increased		3		0
Mean cell haemoglobin concentration increased		2		0
Mean cell haemoglobin increased		1		0
Mean cell volume increased		1		0
Monocyte count decreased		6		0
Monocyte count increased		8		0
Neutrophil count decreased		1		0
Neutrophil count increased		17		0
Platelet count increased		1		0
Prostatic specific antigen increased		1		0
QRS axis abnormal		1		0
Red blood cell count increased		1		0
Red cell distribution width increased		3		0
White blood cell count decreased		2		0
White blood cell count increased		19		0
**Metabolism and nutrition disorders**	**4 (36.4%)**	**5**	**2 (12.5%)**	**2**
Abnormal weight gain		1		0
Diabetes mellitus		1		0
Hypercalcaemia		2		0

Hypercholesterolaemia		0		1
Hyperglycaemia		0		1
Hyperuricaemia		1		0
**Musculoskeletal and connective tissue disorders**	**7 (63.6%)**	**15**	**11 (68.8%)**	**16**
Arthralgia		4		1
Arthritis		0		4
Back pain		1		7
Bone pain		0		2
Limb discomfort		1		0
Muscle spasms		1		0
Muscular weakness		2		0
Musculoskeletal pain		0		1
Myalgia		1		0
Osteoarthritis		1		0
Pain in extremity		2		0
Periarthritis		1		0
Rotator cuff syndrome		1		0
Tendon disorder		0		1
**Neoplasms benign, malignant and unspecified (incl cysts and polyps)**	**1 (9.1%)**	**1**	**0 (0%)**	**0**
Keratoacanthoma		1		0
**Nervous system disorders**	**8 (72.7%)**	**36**	**6 (37.5%)**	**7**
Cognitive disorder		1		0
Dyskinesia		4		0
Freezing phenomenon		0		1
Haemorrhage intracranial		2		0
Headache		3		0

Hyperkinesia		1		0
Hypersomnia		0		1
Memory impairment		2		1
Nervous system disorder		0		1
Neuropathy peripheral		3		0
Paraesthesia		2		0
Parkinsonian gait		2		0
Parkinsonism		12		2
Sciatica		0		1
Tremor		4		0
**Psychiatric disorders**	**8 (72.7%)**	**14**	**3 (18.8%)**	**4**
Anxiety		4		0
Apathy		0		1
Confusional state		2		0
Depressed mood		1		0
Depression		0		1
Dopamine dysregulation syndrome		1		0
Hallucination		1		0
Insomnia		0		1
Mental disorder		0		1
Psychotic disorder		1		0
Rapid eye movement sleep behaviour disorder		2		0
Sleep disorder		1		0
Stress		1		0
**Renal and urinary disorders**	**1 (9.1%)**	**2**	**1 (6.3%)**	**1**
Incontinence		1		0
Micturition urgency		0		1

Pollakiuria		1		0
**Reproductive system and breast disorders**	**1 (9.1%)**	**2**	**1 (6.3%)**	**1**
Erectile dysfunction		1		0
Genital pain		0		1
Testicular disorder		1		0
**Respiratory, thoracic and mediastinal disorders**	**1 (9.1%)**	**1**	**1 (6.3%)**	**1**
Cough		1		1
**Skin and subcutaneous tissue disorders**	**4 (36.4%)**	**7**	**2 (12.5%)**	**5**
Erythema		1		0
Hyperhidrosis		1		1
Nail discolouration		1		0
Rash		0		3
Rash erythematous		2		0
Skin hyperpigmentation		1		1
Skin ulcer		1		0
**Surgical and medical procedures**	**0 (0%)**	**0**	**2 (12.5%)**	**2**
Plastic surgery		0		1
Tooth extraction		0		1
**Vascular disorders**	**4 (36.4%)**	**8**	**2 (12.5%)**	**2**
Hot flush		1		0
Hypertension		6		2
Hypotension		1		0

[1] Numbers presented in this table exclude events which are also reported as serious adverse events (presented in Supplementary Tables 4a and 4b)

[2] Participants are only counted once within each organ system

[3] Total number of incidences are presented as each individual report (even if reported within a single participant)

Supplementary Table 4a: Serious Adverse Events Table: Control Group

Event	Status	Comments	MedDRA preferred term (PT)	MedDRA system organ class (SOC)
Cervical disc herniation	Resolved	After surgery	Intervertebral disc protrusion	Musculoskeletal and connective tissue disorders
Lumbar disc herniation	Resolved	After surgery	Intervertebral disc protrusion	Musculoskeletal and connective tissue disorders
Rectal cancer	Resolved	After surgery	Rectal cancer	Neoplasms benign, malignant and unspecified (incl cysts and polyps)
Ileostomy closure	Recovered		Ileostomy	Surgical and medical procedures
Urinary tract infection	Recovered	Urosepsis requiring hospitalisation, treated with IV antibiotics	Urinary tract infection	Infections and infestations

Supplementary Table 4b: Serious Adverse Events Transplant Group

Event	Status	Comments	MedDRA preferred term (PT)	MedDRA system organ class (SOC)
Episode of supraventricular tachycardia (SVT)	Resolved	Terminated with IV beta blockers + cardiac ablation	Supraventricular tachycardia	Cardiac disorders
Haemorrhage in the right frontal lobe	Resolved	Post-transplant procedure. Observation and prolonged hospital stay	Haemorrhage intracranial	Nervous system disorders
Vomiting	Resolved	Resolved following reduction in dose of the immunosuppressive drugs.	Vomiting	Gastrointestinal disorders
Intracerebral haemorrhage	Resolved	Post-transplant procedure. Observation and prolonged hospital stay	Haemorrhage intracranial	Nervous system disorders
Kaposi's sarcoma	Resolved	Developed while on immunosuppression. Disappeared when immunotherapy discontinued.	Kaposi's sarcoma	Neoplasms benign, malignant and unspecified (including cysts and polyps)
Wedge pulmonary embolism	Resolved	Started on anticoagulation	Pulmonary embolism	Vascular disorders
Wound dehiscence	Resolved	Wound dehiscence	Wound dehiscence	Injury, poisoning and procedural complications

Supplementary Table 5: Inclusion and exclusion criteria for patients in the observational TransEuro study. Additional criteria for the cohort approached for the transplant trial are highlighted in bold.

<u>Inclusion criteria.</u>

- PD as defined by the Queen Square Brain Bank criteria
- Disease duration **≥2 yr and ≤13 yr**
- Aged ≥30 yr and **≤68 yr** at the time of grafting
- Hoehn & Yahr stage 2 or better when in ON medication state
- On no therapy or only receiving standard anti-PD treatment
- No significant L-dopa-induced dyskinesia
- **Significant ≥33% improvement in their UPDRS part III motor score in response to an acute dose of L-dopa**
- **Preserved** [^{18}F]-fluorodopa **signal in ventral striatum**

<u>Exclusion criteria.</u>

- Atypical parkinsonism, including [^{18}F]-fluorodopa PET patterns consistent with this
- Mini mental state examination (MMSE) score of <26 or evidence for dementia using DSM-IV criteria
- Unable to copy normally and accurately two interlocking pentagons and a semantic fluency score of <20 over 90 s
- Ongoing major medical or psychiatric disorder, including depression and psychosis
- Other concomitant treatment with neuroleptics

• Significant drug-induced dyskinesia (>2 for any body part on the AIMS scale)

• Previous neurosurgery

• Unable to be imaged using MRI

• **Clinically insignificant response to L-dopa**

• **Any contraindication to immunosuppression therapy**

• **Patients on anticoagulants**

• **Patients who are left-handed**

Supplementary Table 6: The assessment protocol undertaken for the patients.

All visits:

- Unified Parkinson’s Disease Rating Scale (UPDRS) Part III (OFF and ON Medications)
- RUSH Dyskinesia Scale (OFF and ON Medications)
- Abnormal Involuntary Movement Scale (OFF and ON Medications)
- 30 Second Tap Test, 9 Hole Pegboard, Timed Sit-stand-walk Test (OFF and ON Medications)
- UPDRS Parts I, II and IV
- Addenbrooke’s Cognitive Examination-Revised Edition (ACE-R)
- Beck Depression Inventory
- Questionnaire for Impulsive-Compulsive Disorders in Parkinson's Disease–Rating Scale (QUIP-RS)
- Parkinson’s Disease Diaries

Additional tests undertaken at annual (12/24/36 months etc.) visits only

- Hopkins Verbal Learning Test-4
- Stroop (Colour, Word & Interference)
- Digit Span

- Verbal and Categorical Fluency
- Graded Naming Task
- WAIS-Similarities
- PD Sleep Scale
- Apathy Evaluation Scale
- Parkinson’s Disease Questionnaire-39 (PDQ-39)
- EQ-5D-5Level
- Quality of Life Scale
- CANTAB One Touch Stockings of Cambridge (OTS), Reaction Time (RTI) and Recognition Memory Batteries
- PD Non-Motor Symptom Scale
- Screening Questionnaire for Compulsive Behaviours

Supplementary Figure 1: Landmarks used for the dissection of the VM tissue used for grafting. These were used by all sites and documented via photographs that were then viewed by the other sites to ensure consistency in dissection across sites.

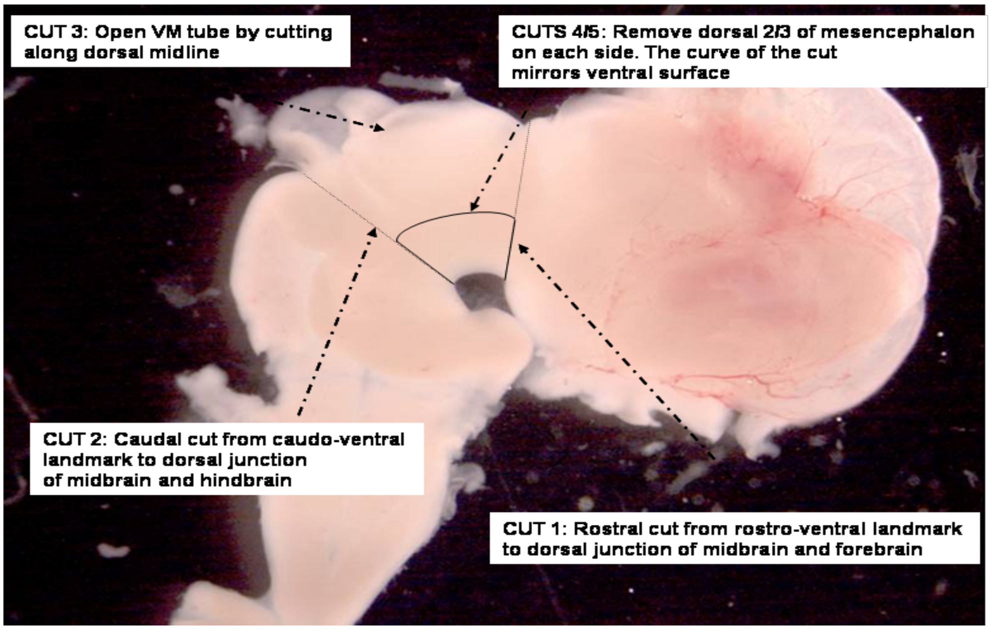

Supplementary Figure 2: T2 SWI 1mm MRI scans done in the immediate post-operative period showing the needle tracts within the putamen after each of the operations in one of the transplanted patients (courtesy of Dr Hjalmar Bjartmarz).

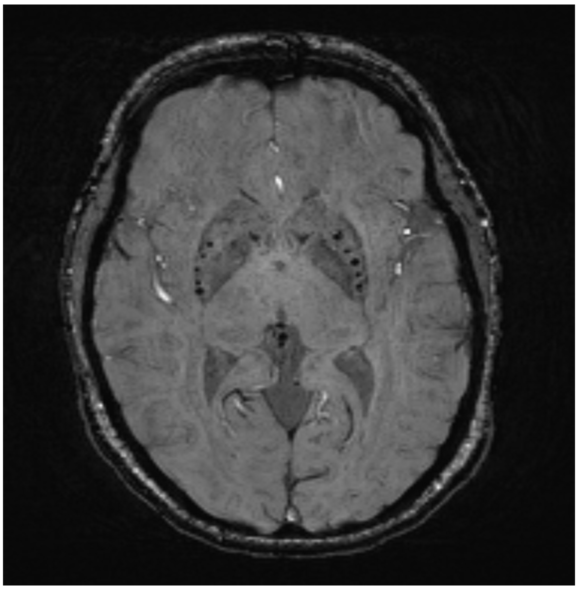

Supplementary Figure 3: Correlation between actually scored UPDRS and the score given by the rater blinded to the patient in the video

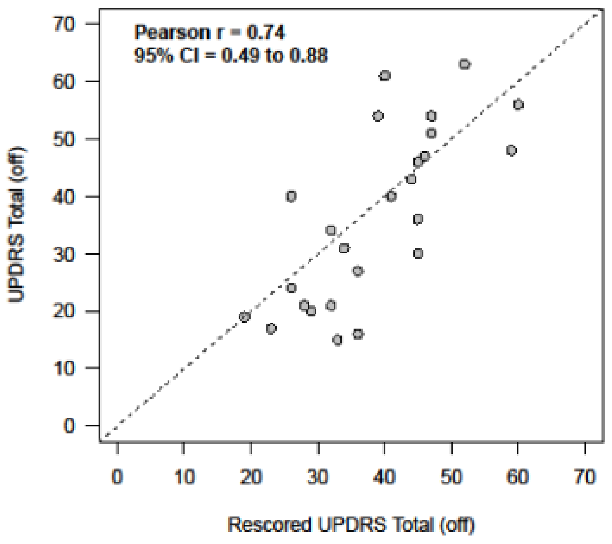

Supplementary Figure 4

Fig_S4a: [^{18}F]FDOPA: [^{18}F]FDOPA PET for bilaterally transplanted patients who completed imaging at pre- and post-transplantation. Parametric images (K_i) are overlaid onto patient T1-weighted MPRAGE scans rigidly aligned to the MNI template and displayed on representative sagittal slices covering the putamen of both right and left hemispheres. OFF-state UPDRS-III scores (*U*) from assessments closest in time to the PET acquisitions are displayed.

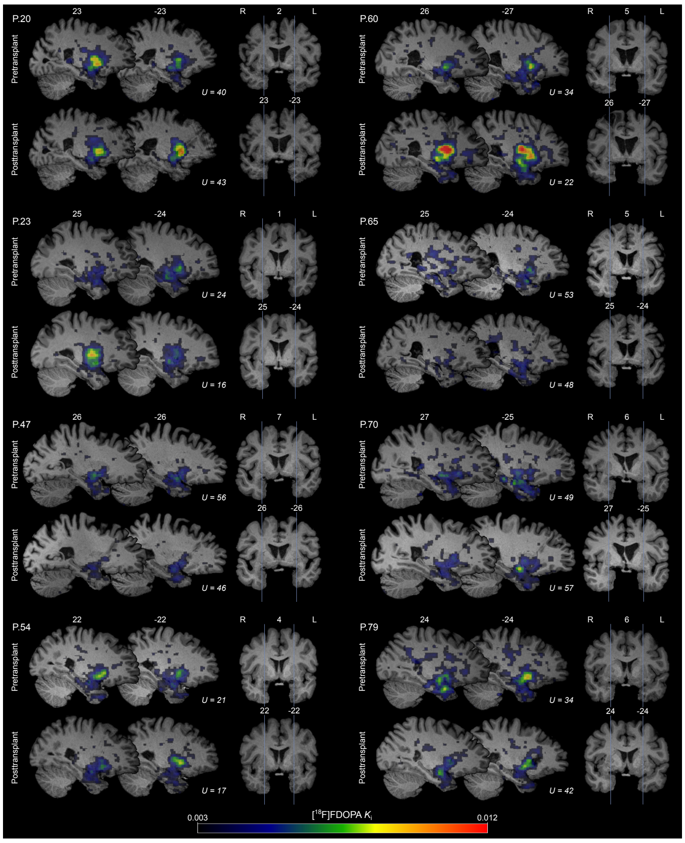

Fig S4b:_ [^{11}C]PE2I: [^{11}C]PE2I PET for bilaterally transplanted patients who completed imaging at pre- and post-transplantation. Parametric images (BP_{ND}) are overlaid onto patient T1-weighted MPRAGE scans rigidly aligned to the MNI template and displayed on representative sagittal slices covering the putamen of both right and left hemispheres. OFF-state UPDRS-III scores (*U*) from assessments closest in time to the PET acquisitions are displayed.

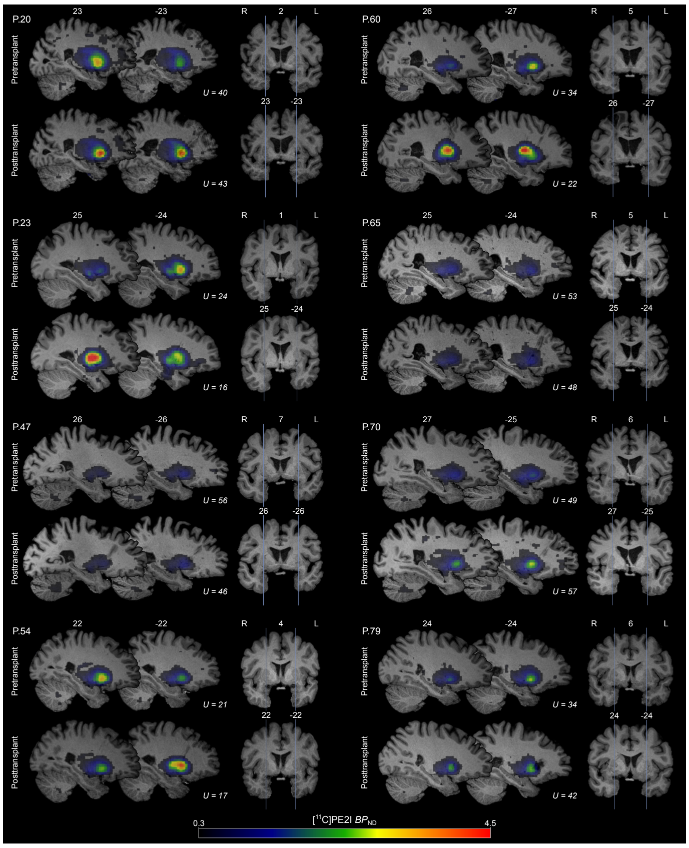

An Open Label Study to Assess the Safety and Efficacy of Neural Allo-Transplantation with Fetal Ventral Mesencephalic Tissue in Patients with Parkinson's disease

TRANSEURO

REC No: **12/EE/0096**

Chief Investigator:

Professor Roger Barker

Address: Cambridge Centre for Brain Repair

E.D. Adrian Building

Forvie Site, Robinson Way

Cambridge

CB2 0PY

Uk

Tel: 01223 331160

Fax: 01223 331174

Email: dmj34@cam.ac.uk

Joint Study Sponsor:

Cambridge University Hospitals NHS Foundation Trust and

University of Cambridge

Trial Coordination:

Cambridge Centre for Brain Repair

E.D. Adrian Building

Forvie Site

Robinson Way

Cambridge

CB2 0PY

UK

Telephone: 01223 331160

Fax:01223 331174

Approvals Signature Page

Professor Roger Barker

Chief Investigator

Changes to any personnel listed above will not be classed as a substantial amendment, but will be documented with a note to file.

TABLE OF CONTENTS

1 ABBREVIATIONS 28
2 INVESTIGATORS 30
3 INVESTIGATOR STATEMENT 31
4 Summary 32
INTRODUCTION 35
4.1 Background 35
4.2 Study rationale 36
5 Objectives 38
5.1 Primary Objective 38
5.2 Secondary Objective 38
5.3 Primary Endpoints 38
5.4 Secondary Endpoints 38
5.5 Safety Endpoints 38
6 Study Design 39
6.1 Study Sites 39
6.2 Study Population 39
7 PATIENT SELECTION 39
7.1 Inclusion Criteria 39
7.2 Exclusion Criteria 39
7.3 Removal and replacement of patient from the study or study treatment 40
8 Treatment regimen, expected toxicity and dose modifications 40
8.1 Treatment Schedule 40
8.1.1 Pre Treatment 40
8.1.2 Hospitalisation 41
8.1.3 Anaesthesia 41
8.1.4 Head Frame 41
8.1.5 Surgical Device and tissue administration 41
8.1.6 Fetal Tissue Preparation 41
8.1.7 Medication 42
8.1.8 Preparation of surgical site 42
8.1.9 Selection of site for tissue administration 42
8.1.10 Microbiology and Virology testing 43
8.2 Concomitant Medication and Treatment 43
9 Schedule of Investigations and Evaluations 43
9.1 Screening Visit 43
9.1.1 For those patients NOT currently taking any dopaminergic medication 43
9.1.2 PET Imaging 44
9.1.3 Baseline Visit 45
9.1.4 Day before Surgery 46
9.1.5 Day of Surgery 46

9.1.6 Follow Up Procedures for the first 12, 24 and 48 hours post surgery 46
9.1.7 Follow up procedures 7, 14, 21, 28 and 42 days post surgery 47
9.1.8 Follow up procedures 2, 3, 4, 5, 6, 9 and 12 months post surgery 47
9.2 Other Study-related visits 47
9.3 Schedule of assessments 48
10 Assessment of Safety 50
10.1 Reporting procedure 51
10.1.1 Adverse Event Reporting and Treatment 51
10.1.2 Pre-existing conditions 51
10.1.3 Diagnostic and Surgical Procedures 52
10.1.4 Follow-up of Adverse Events and Clinically Relevant Laboratory Abnormalities 52
10.2 Serious Adverse Event and SUSAR Reporting Procedure 52
10.2.1 Expected side effects –These AEs must be reported as SAEs if they fulfil any other SAE criteria. 52
10.2.2 Reporting of SAEs 52
10.2.3 Follow-up of SAEs 54
11 Statistics and sample size 54
11.1 Statistical Power 54
11.2 Statistical analysis 54
12 Study Management 55
12.1 Investigator authorisation procedure 55
12.2 Patient registration procedure 55
12.3 Case Report Forms and procedures for collecting data 55
12.3.1 Site monitoring 55
12.3.2 Case Report Form Procedures 56
12.4 Essential Documentation and Archiving 59
12.5 Amendments 59
12.6 Trial sponsorship and financing 60
12.7 Trial insurance 60
13 Ethical Considerations 60
13.1 Patient protection 60
13.2 Informed consent 61
13.2.1 In the event of consent documentation requiring translation 61
14 Publication Policy and Press Releases 61
15 References 62

ABBREVIATIONS

AE	Adverse Event
AIMS	Abnormal Involuntary Movements Scale
AR	Adverse Reaction
ATC	Autonomic Therapeutic Chemical
BRC	Cambridge Centre for Brain Repair
CMV	Cytomegalovirus
CRF	Case Report Form
CRP	C-reactive protein
CV	Curriculum Vitae
Cy A	Cyclosporin A
DA	Dopamine
DMEM	Dulbecco's modified eagle medium
DTI	Diffusion Tensor imaging
eCRF	Electronic Case Report Form
EMEA	European Medicines Evaluation Agency
EU	European Union
FBC	Full Blood Count
FDA	Food and Drugs Administration (USA)
F-Dopa	Fluoro-dopa
fMRI	Functional Magnetic Resonance Imaging
GID	Graft Induced Dyskinesia's
GMP	Good Manufacturing Practice
GP	General Practitioner
HBV	Hepatitis B Virus
HCV	Hepatitis C Virus
HIV	Human Immunodeficiency Virus
ICH-GCP	International Conference on Harmonisation – Good Clinical Practice
IEC	Independent Ethics Committtee
IV	Intra Venous
LID	Levodopa Induced Dyskinesia

MedDRA	Medical dictionary for regulatory activities
MMSE	Mini Mental State Exam
MR	Magnetic Resonance
MRI	Magnetic Resonance Imaging
NIH	National Institute of Health
NHS	National Health Service
PCR	Polymerase Chain Reaction
PD	Parkinson’s disease
PET	Positron Emission Tomography
QC	Quality Control
REC	Research Ethics Committee
RSN	Resting State Networks
SAE	Serious Adverse Event
SAR	Serious Adverse Reaction
SNc	Substantia Nigra Pars Compacta
SOP	Standard Operating Procedures
SUSAR	Suspected Unexpected Serious Adverse Reaction
TPMT	Thiopurine Methyltransferase
UPDRS	Unified Parkinson’s Disease Rating Scale
VM	Ventral Mesencephalic
WMA	World Medical Association

INVESTIGATORS

Trial Development Group

Roger Barker, Colin Watts	Department of Clinical Neurosciences, Addenbrooke's Hospital, University of Cambridge
Hakan Widner, Stig Rehncrona	Lund University, Sweden
Paola Piccini, Marios Politis	Imperial College, London
Tom Foltynie	University College London
Guido Nikkhah, Christian Winkler	Universitaetsklinikum Freiburg

INVESTIGATOR STATEMENT

Principle Investigator signature:

I have read and agree to the protocol, as detailed in this document. I am aware of my responsibilities as an Investigator under the guidelines of Good Clinical Practice (ICH-GCP) the Declaration of Helsinki, the applicable regulations of the relevant NHS Trusts and the study protocol and I agree to conduct the study according to these guidelines and to appropriately direct and assist the staff under my control, who will be involved in the study.

Principal Investigator's Name: ______________________________

Signature: ______________________________

Date: ______________________________

Summary

Title	An Open Label Study to Assess the Safety and Efficacy of Neural Allo-Transplantation with Fetal Ventral Mesencephalic Tissue in Patients with Parkinson's disease
Clinical Phase	Phase 1
Medical condition or disease under investigation	Parkinson's Disease (PD)
Primary objective	To measure the safety and feasibility of neural transplantation in the treatment of patients with Parkinson's disease as assessed using standard surgical, neurological, psychiatric and psychometric testing including the incidence and severity of 'off-medication' / graft induced dyskinesias.
Secondary objective(s)	To assess the clinical efficacy following neural transplantation of foetal VM tissue.
Study design	This is a single centre, open label, transplantation study (see Appendix 1)
Primary Endpoints	• The change in motor UPDRS in a defined "OFF" state at 36 months post transplantation. "OFF" being defined as receiving no dopamine (DA) therapy for 12 hours prior to assessment or longer in the case of long acting dopamine agonists (e.g. Ropinirole slow release).
Secondary Endpoints	• Change in timed motor tasks at 36 months post transplantation<br>• The number of patients with dyskinesias (including L-dopa and graft induced dyskinesias) at 36 months post transplantation.<br>• L-dopa equivalence medication doses at 36 months post transplantation.<br>• Number of patients on L-dopa therapy at 36 months post transplantation.<br>• The amount of 'off' time 36 months post transplantation.<br>• Quality of life as assessed by PDQ-39 and calculated "overall outcome changes" 36 months post transplantation.<br>• Changes in F-DOPA PET in transplanted patients 36 months post transplantation.
**N.B: All efficacy assessments are conducted as part of the TRANSEURO observational study (10/0304H/77) with the exception of the outcomes of the PET scans.**	

Safety Endpoints	• The number of adverse events and serious adverse events associated with the neural transplant.<br>• Laboratory Parameters – any reported changes in haematology, biochemistry or urinalysis measures outside the normal range.<br>• Other Safety parameters – vital signs, Physical Exam – new abnormalities will be recorded as an adverse event.
Study Population	40 Patients will be recruited to this open label study from the TRANSEURO observational study (10/0304H/77). All 40 patients will be assigned to receive PET imaging and from this cohort 20 patients will be randomly assigned to receive transplantation. The remaining 20 will be assigned to be a comparison group of controls who will receivethe same observational and scanning assessments but WILL NOT receive any surgical procedures.
Summary of eligibility criteria	**<u>Inclusion Criteria</u>**<br>Patients must meet ALL of the following criteria to be considered for the enrolment into this study:<br>• PD as defined using Queen's Square Brain Bank criteria.<br>• Disease duration ≥ 2 years and ≤ 13 years.<br>• Aged ≥ 30 years and ≤ 68 years at the time of grafting.<br>• Hoehn & Yahr stage 2 or better when 'on'.<br>• On standard anti PD medication without significant LIDs defined as a score of ≤2 on the AIMS dyskinesia rating scale, in any body part.<br>• Patients must be right handed.<br><br>**<u>Exclusion Criteria</u>**<br>Any of the following will exclude patients from being enrolled in the study:<br>• Atypical or secondary parkinsonism including F-DOPA PET patterns consistent with this.<br>• Clinically insignificant response to Levodopa (as evaluated by the clinician) and/or apomorphine challenge.<br>• Mini-Mental State Examination (MMSE) score of less than 26.<br>• Unable to do normal copying of interlocking pentagons and semantic fluency score for naming animals of less than 20 over 90

	seconds as these have recently been associated with the earlier onset of dementia in PD.<br>• Ongoing major medical or psychiatric disorder including depression and psychosis.<br>• Other concomitant treatment with neuroleptics (inc. Atypical neuroleptics) and cholinesterase inhibitors.<br>• Significant drug induced dyskinesia defined as a score of >2 on the AIMS dyskinesia rating scale, in any body part.<br>• Previous neurosurgery, cell therapy or organ transplantation.<br>• Unable to be imaged using MRI.<br>• Any contraindication to immunosuppression therapy.<br>• Patients on anticoagulants<br>• Patients who are left handed
Statistical Analysis	The study will be analysed using a variety of descriptive statistics and exploratory figures by a biostatistician independent of the research team.
Procedures:	See section 10 Schedule of Investigations and Evaluations
Procedures for safety monitoring during trial	SAE reporting, See Section 11

INTRODUCTION

Background

Parkinson's disease (PD) is a common neurodegenerative disease affecting approximately 1% of people over 65 years of age, and as such is likely to become more common as the population ages and lives longer. It is characterised clinically by the development of bradykinesia, rigidity and a resting tremor, which has been attributed in part to the progressive degeneration of the dopaminergic input from the substantia nigra to the striatum. The cause for this progressive loss of nigrostriatal dopaminergic neurons is not entirely understood, but it is associated with the presence of alpha-synuclein positive Lewy bodies, in some of the remaining substantia nigra pars compacta neurons (SNc) at post-mortem. However, Lewy bodies are also found in many other parts of the brain (including a range of subcortical and brainstem nuclei as well as the cortex) and this may underlie the non-motor features of PD such as cognitive, sleep, and affective problems as well as autonomic dysfunction, which are found in the majority of cases of PD. Indeed, it is increasingly being understood that PD is a disorder which has widespread pathology from its onset and that therefore the nigral pathology is only part of a much more diffuse pathological process [1, 2]. However whilst this is true, the core loss of the dopaminergic nigrostriatal pathway is not disputed, nor the effects of treatment targeting this pathway in the clinic.

Thus, despite this non-nigral, non-dopaminergic pathology, the principal feature of the disease remains the progressive loss of dopamine (DA) in the striatum, which can be treated successfully with a range of symptomatic dopaminergic drug therapies. Thus both levodopa and DA agonists significantly help many of the features of PD, particularly in the early stages of the disease [3]. However with time, the symptoms progress and this coupled to the long term use of dopaminergic drug therapies produces a range of problems including the development of drug induced motor complications such as 'on-off' fluctuations and levodopa-induced dyskinesias (LID).At this stage of the disease, drug therapies become increasingly disappointing in terms of a reliable therapeutic benefit [4]. Therefore other therapeutic approaches are used, including more invasive ways of delivering more continuous dopaminergic therapy, such as apomorphine pumps and DuoDopa® (constant delivery of L-Dopa into the small bowel), as well as neurosurgical interventions such as deep brain stimulation, especially of the subthalamic nucleus [5].

These latter therapies can be very effective, but only ever treat the symptoms without any attempt to repair the underlying diseased network. Thus these treatments also start to fail, in part because of the continued loss of nigral dopaminergic neurons [6,7]. Therefore, whilst an improved understanding of disease pathogenesis may enable us to better treat all aspects of PD, more restorative approaches to repairing the dopaminergic nigrostriatal tract, including cell replacement, neurotrophic support and pharmacological and gene therapies, may also prove very useful [8, 9].

One of the most effective reparative therapies in patients to date has been with allotransplants of DA neuroblasts obtained from fetal ventral mesencephalic (VM) tissue. However, this cell transplantation approach has given inconsistent results. Some grafted patients have responded extremely well and have come off anti-PD medication for years, whilst others have shown no or only modest clinical improvements [10-15]. Moreover, a subset of patients also developed severe, off-state graft-induced dyskinesias (GID), which in a few cases have required additional neurosurgical intervention [16-18]. The reasons behind this heterogeneity of outcomes and the emergence of graft-induced dyskinesias (GIDs) in particular, need to be better understood, not least in the perspective of the rapid advances

that are now being made in the development of stem-cell based therapies. There is therefore an urgent need to revisit the trials that have already been done with fetal VM tissue in PD patients, with the expectation that a critical reassessment can form the basis for an optimised and more standardised procedure that will translate into more consistently efficacious transplants with minimal side –effects. This is important despite the recent publications showing Lewy Body pathology can develop after a number of years in some of the grafted dopaminergic neurons, as even in these cases the transplants were still functional and the majority of grafted cells did not display such pathology [16-18]. Thus whilst of great interest and significance to the development of all cell based therapies for PD, it does not undermine the strategy that began 20 years ago with VM tissue transplants given the clear long term benefits that have been reported [19].

Study rationale

Clinical trials of cell therapy in PD patients were first performed in Lund in the late 1980s, followed by a number of similar, small trials in other European and North American centres. These initial studies performed on small groups of advanced PD patients were all open label but firmly established safety of the procedure. The results obtained in these trials have shown that the grafted dopaminergic [DA] neurons can survive and function long-term, over more than 10 years, and that some patients have shown clear clinical benefits, especially with respect to their bradykinesia and rigidity, with reductions in L-dopa requirements. Using functional imaging it has also been shown that the grafted DA neurons can restore striatal DA release and provide a sustained re-activation of motor cortical areas, i.e. key areas that were underactive prior to grafting. Post-mortem studies have shown excellent long-term survival of the grafted DA neurons, notwithstanding the observation that some of the long-term surviving transplants (at 12-15 years after grafting) have now been shown to contain signs of PD-related pathologies, i.e. neuronal Lewy-bodies and alpha-synuclein positive inclusions in the grafts. However, such changes have been observed only in some and not in all patients and when seen the extent of the pathology is limited to a small number of the grafted DA cells and the clinical consequence, if any, not known.

However, the outcomes of two NIH sponsored double blind placebo controlled trials, which published their main findings in 2001 and 2003, have raised major concerns. In both these trials the grafted patients did not show any significant improvement overall compared to sham-operated controls at 1 and 2 years post grafting. Furthermore, a significant number of patients in both trials developed GIDs, which in some cases were so severe that further neurosurgery was needed to remedy the situation.

The reasons for the variable, and overall poor, outcome in these trials, including the generation of GIDs, have been the subject of much debate but have recently centred on three key elements, with an additional possible fourth element:

- The selection of patients in terms of clinical phenotype, disease stage and pattern of striatal dopaminergic denervation at the time of grafting;
- The differences in immunosuppressive regimes and the risk that incomplete immunosuppression in combination with the use of solid graft methods may lead to the development of detrimental immune/inflammatory reactions at the graft site with compromise of the grafted dopamine cell function;
- The mode of engraftment and differences in graft cell survival, and the risk for inhomogenous delivery of dopaminergic neurons and the generation of potentially dyskinesia-inducing "patchy" innervations in the host striatum;
- A final possible element is the composition of the grafted tissue and the ratio of serotoninergic to dopaminergic neurons within the graft. There is emerging evidence that serotoninergic neurons can release dopamine in a relatively unregulated fashion given they lack transporters for it, and as such may use L-dopa as a false transmitter which may not only underlie the development of L-dopa induced dyskinesias but may also contribute to GIDs.

Failure of the NIH trials to demonstrate any overall clinical benefits in the grafted patients, and the unexpected and worrisome development of GIDs in a significant number of patients in these trials has represented a major hurdle for the future development of cell based therapies for PD and it is in this and related areas that this project seeks to move the field forward and go beyond the current state of the art for this treatment approach.

This project has gathered together all the available expertise in this area to resolve or reduce the risk of the previous complications seen with VM transplants in patients with PD. We will conduct a new round of clinical trials, involving a step-by-step optimisation of all technical aspects of the grafting procedure and patient selection and assessment, in order to improve clinical efficiency and consistency, in the absence of troublesome dyskinesias.

Objectives

Primary Objective

- To measure the safety and feasibility of neural transplantation in the treatment of patients with Parkinson's disease as assessed using standard surgical, neurological, psychiatric and psychometric testing including the incidence and severity of 'off' / graft induced dyskinesias.

Secondary Objective

- To assess the clinical efficacy following neural transplantation of foetal VM tissue.

Primary Endpoints

- The change in motor UPDRS in a defined "OFF" period at 36 months post transplantation. "OFF" being defined as receiving no dopamine (DA) therapy for 12 hours prior to assessment or longer in the case of long acting dopamine agonists (e.g. Ropinirole slow release).

NB: Efficacy measures are recorded as part of the TRANSEURO observational study (10/0304H/77) and are therefore not repeated in this protocol.

Secondary Endpoints

- Change in timed motor tasks at 36 months post transplantation
- The number of patients with dyskinesias (including L-dopa and graft induced dyskinesias) at 36 months post transplantation.
- L-dopa equivalence medication doses at 36 months post transplantation.
- Number of patients on L-dopa therapy at 36 months post transplantation.
- The amount of 'off' time 36 months post transplantation.
- Quality of life assessed by PDQ-39 and calculated "overall outcome changes" 36 months post transplantation.
- Changes in F-DOPA PET in transplanted patients 36 months post transplantation.

NB: Efficacy measures are recorded as part of the TRANSEURO observational study (10/0304H/77) and are therefore not repeated in this protocol with the exception of the PET scans.

Safety Endpoints

- The number of adverse events and serious adverse events associated with the neural transplant.
- Laboratory Parameters – any reported changes in haematology, biochemistry or urinalysis measures outside the normal range.
- Other Safety parameters – vitals, Physical Exam – new abnormalities will be recorded as an adverse event.

Study Design

Study Sites

This is a single centre, open label PET imaging and transplantation study to be based at Cambridge University Hospitals, NHS Foundation Trust, Cambridge. Cambridge has extensive experience in neural transplantation. PET imaging will be conducted at Imanova, Hammersmith Hospital, London. Patients will be recruited from the Transeuro observational study (Rec: 10/H0304/77) where patients are recruited from a number of sites, University College London, Imperial College London, Cambridge and Cardiff.

Other EU sites (Freiburg, Germany & Lund, Sweden) will be conducting a comparable study in parallel (Appendix 1) adopting the same methodology described in this protocol however they will be obtaining independent sponsorship and regulatory approval.

Study Population

Up to forty patients will be entered into this study in Cambridge; they will be compared with the remaining participants from the TRANSEURO observational study (REC 10/H0304/77) who were not selected. All 40 patients will undergo PET imaging at Imanova for baseline scanning. From these 40 patients,   20 will be randomly selected to undergo neural transplantation. The 20 patients who are not selected for neural transplant will remain in the PET imaging arm of this study and undergo scans at 18 and 36 months along with their ongoing observational study visit. All patients will be consented as per ICH guidelines prior to any protocol specific procedures. For patients who are delayed in the study, they will be scanned at Baseline, baseline and 18 months.

The expected recruitment period is 18 months.

PATIENT SELECTION

Inclusion Criteria

Patients must meet ALL of the following criteria to be considered for enrolment into this study:

- PD as defined using Queens Square Brain Bank criteria.
- Disease duration ≤ 2 years and ≤ 13 years.
- Aged ≥ 30 years and ≤ 68 years at the time of grafting.
- Hoehn & Yahr stage 2 or better when 'on'.
- On standard anti PD medication without significant LIDs as defined by a score of >2 in the AIMS dyskinesias rating scale, in any body part.
- Patients must be right handed.

Exclusion Criteria

The patient will be excluded from the study if they meet any of the below criteria:

- Atypical or secondary parkinsonism including F-DOPA PET patterns consistent with this.
- Clinically insignificant response to Levodopa (as evaluated by the clinician) and/or apomorphine challenge.
- Mini-Mental State Examination (MMSE) score of less than 26.
- Unable to do normal copying of interlocking pentagons and semantic fluency score for naming animals of less than 20 over 90 seconds as these have recently been associated with earlier onset dementia in PD.
- Ongoing major medical or psychiatric disorder including depression and psychosis.
- Other concomitant treatment with neuroleptics (inc. Atypical neuroleptics) and cholinesterase inhibitors.
- Significant drug induced dyskinesias as defined by a score of >2 in the AIMS dyskinesias rating scale, in any body part.
- Previous neurosurgery, cell therapy or organ transplantation.
- Unable to be imaged using MRI.
- Any contraindication to immunosuppressive therapy.
- Any contraindication to neurosurgery.
- Patients who are left handed.

Removal and replacement of patient from the study or study treatment

A patient can withdraw consent from any study related procedure at any time during the study and this will not affect their standard of care.

Any patient who withdraws prior to surgery will be replaced to ensure 20 patients are transplanted.

Any patient who withdraws post surgery will be ask for consent to contact their neurologist/geriatrician to allow us to monitor for the emergence of any potential side effects of the surgery.

Treatment regimen, expected toxicity and dose modifications

Treatment Schedule

Pre Treatment

Patients will attend their local centre for their observational study visit where they will complete their usual tasks as outlined in protocol ref 10/H0304/77. The opportunity to take part in this imaging and transplant study will be discussed in detail with the patient and they will be given the Patient Information Sheet and the opportunity to ask any questions they may have. Patients are under no obligation to take part in the transplant trial. Once they have completed the visit, the patient will be free to go home and discuss the option of a transplant with friends and family. A follow up phone call will be made and the opportunity to come back to clinic for further discussion of the transplantation procedure

will be offered. Once the patient has decided they wish to take part in this study, they will be invited to attend their local hospital where they will sign informed consent and they will complete the screening process. Once a patient has completed the screening visit, they will be booked for their baseline PET scan at Imanova. Following successful completion of the PET scan a date will be set for patients to return for the baseline visit at Addenbrooke's hospital, Cambridge. If more than 3 months has passed since their 12 month visit, the patient will be required to complete the same tasks they would do in their 12 month observational visit prior to transplantation. At this visit, the clinician will run through what to expect on the day of surgery, their medication will be reviewed and it will be determined which medications, if any, should be withdrawn prior to surgery and when this should happen. Patients will also be provided with the contact details for the team in Cambridge.

Hospitalisation

Patients will be admitted to Addenbrooke's hospital the day before their planned transplant. On the baseline visit preceding surgery it must be determined which medication has to be withdrawn. No oral food is allowed within 8 hours, and no drinking within 6 hours prior to surgery.

Anaesthesia

Surgery is performed under general anaesthesia. The patient will be intubated and ventilated. This is preferable due to the long time of the surgical procedure. The actual use of anaesthetic will depend on the local standards of Addenbrooke's Hospital.

Head Frame

The standard head fixation system from Addenbrooke's Hospital will used.

Surgical Device and tissue administration

The surgical device to be used in the transplant is The Rehncrona – Legradi Transplantation Instrument. The procedure will be a bilateral transplant (assuming sufficient tissue is available for grafting, otherwise the patient will undergo sequential, unilateral transplants with the minimum time between transplants) using image guided targeting, with burr hole size of 14mm. Sulci/ventricles will be avoided using standard stereotactic techniques for image guided stereotactic surgery and trajectory planning. To prevent any brain shift, burr hole irrigation will be performed along with head up tilt of around 30-45 degrees. Five to seven trajectories per putamen will be made. This will be calculated during surgery based on the images of each individual putamen with the aim to cover the putamenal volume as completely as possible. In general, there will be two trajectories into the frontal part (precommissural) and three into the posterior part (postcommissural) giving a total of 5 trajectories (lengths between 9-14 mm). Eight deposits of 2.5 µl each, will be made along each trajectory giving a total of 20µl/trajectory.

Fetal Tissue Preparation

In general, 3-5 foetal VMs will be used for each transplanted putamen and prepared as a crude cell suspension to ensure homogeneous content of each deposit. The cannula will be flushed with the cell suspension prior to insertion along each trajectory to address dead space within the cannula while minimising tissue loss. Each 2.5 µ deposit will be delivered over 15-20 seconds, with a 2 minute waiting

time until retraction of the cannula 1.5 – 1.8 mm, depending of the total length of each planned individual trajectory as calculated from the stereotactic MR images. Eight minutes after the last deposit there will be a slow withdrawal of the cannula and the next trajectory will then be performed.

Medication

Prophylactic antibiotics at the time of surgery and for 48 hours post transplant – Gentamicin 120mg IV and Flucloxacillin 1g, although this may be amended according to updates from the Addenbrooke's Hospital Microbiology department. Osteoporosis prophylaxis will also start at the time of surgery in the form of Alendronic Acid and Calcichew post transplant and gastric lining surgery protection with ranitidine 150mg twice daily or omeprazole 20mg daily.

Immunosuppressive treatment will start before the planned transplantation and will consist of a combination of cyclosporine, 2mg/kg bd (giving serum levels between 100ng/ml and 200ng/ml) to commence the day before transplantation, azathioprine, 2mg/kg per day to commence the day before transplantation and given once per day and prednisolone, 40mg per day (given once a day) starting the day before surgery and reducing thereafter to 5mg by 12 weeks. In addition 1g IV methyl prednisolone will be given at the time of surgery. These oral medications will be continued for at least one year post grafting and maintained with daily doses according to an individual's measurement of blood concentrations of cyclosporine and other haematological and biochemical measures. Oral Septrin will be given as prophylaxis post op, 3 times per week for the duration of the immunotherapy.

CMV negative patients that receive a CMV positive transplant will be prophylactically treated with Valganciclovir 900mg once daily for 3 months. All medication will be dispensed from Addenbrooke's pharmacy and either given to patients at their clinic appointment or sent to the patient at home after their clinic appointment.

Administration of immunosuppressant may increase the risk for cancer slightly if given long term, however as we are only planning to give treatment for a year, then there will be no need for screening for malignancy.

Preparation of surgical site

To avoid contaminations and facilitate the fixation of the stereotactic frame, the patient's head will undergo a complete head shave, if a bilateral transplant is being undertaken. However, if the patient does not wish to have a complete head shave then the procedure can be performed by limiting the shaving to 30-40 mm in the fronto-parietal region.

Selection of site for tissue administration

The transplant site will be selected using stereotactic MRI with frame in situ prior to surgery.

Microbiology and Virology testing

Patients will be tested for HIV, HBV, HCV (all of which are regarded as exclusion criteria). The CMV serology will be tested. Patients will also be tested for Toxo G and HTLV 1. Positive serology for treponema pallidum will be classed as an exclusion criteria.

Concomitant Medication and Treatment

No participation in another clinical treatment trial is allowed. Patients are permitted to take their standard drug therapies during the trial. Concurrent drug therapies must be recorded in the eCRF at the start of the study.

Schedule of Investigations and Evaluations

Screening Visit

Patients will be screened at their local hospital after their 12-month observational study visit has been completed. Having completed the tasks for the 12 month visit, and after signing informed consent, they will complete blood tests and subsequently the PET scan (see 9.1.3) which will ultimately determine if the patient is eligible for a transplant. Other assessments that may be required to be completed following this visit include:

- L-Dopa or Apomorphine challenge **(see details below)**

For those patients NOT currently taking any dopaminergic medication

To demonstrate appropriate alleviation of motor features in response to dopamine as detailed in the exclusion criteria, those patients who are not taking any dopaminergic medication as standard for their PD will be asked to engage in an apomorphine challenge test.

Apomorphine is a directly acting dopamine agonist with no opiate or addictive properties. An apomorphine challenge is useful in assessing the amplitude of motor response in patients with PD and consists of subcutaneous injections of apomorphine, the effects of which last for approximately 90mins during which motor performance will be assessed using standard measures such as the UPDRS. Due to the strong emetic properties of this drug administration will be accompanied by an antiemetic such as domperidone which is started three days before the apomorphine is due to be given.

The following assessments will be completed at their local screening visit to determine eligibility for PET imaging and possible transplantation:

- Patient Registration

- Weight
- Anaesthesia Assessment
- Concomitant medications

- Vital signs – Blood pressure, pulse
- Physical examination
- FBC, CRP, TPMT
- Urea, Creatinine and electrolytes, coagulation screening
- Liver Function tests
- Blood for Immunology – serology / microbiological screening

PET Imaging

Once the patient has completed local screening, their eligibility for recruitment into the PET imaging study will be determined using inclusion & exclusion criteria together with experienced clinical judgment. Forty patients eligible for PET imaging will be selected and an appointment will be made for the patient to attend Imanova for PET scanning. All patients will already have had basic structural MRI imaging under a separate MRI protocol REC Ref: 10/H0805/73 that includes MRI of resting state networks, (RSNs- fMRI), motor task (ME fMRI) and DTI at baseline (0m). This is also to be repeated at 18 and 36 months. Alternatively if patients are delayed, they will have scans at Baseline, baseline and 18 months.

All patients enrolled into the PET imaging arm of the study will undergo a PET protocol consisting of 11C-PE2I, 11C-DASB and 18F-DOPA, at baseline (0m), and then post-transplantation at 18 and 36 months (please see Appendix 1 for full PET protocol). Alternatively if patients are delayed, they will have scans at Baseline, baseline and 18 months. Patients will undergo no more than 3 scanning timepoints.

**PET ligand**	**Biological target**	**Marker**	**Clinical correspondence**
18F-DOPA	Aromatic amino acid decarboxylase (AADC)	Provides measures of AADC activity and allows an indirect measure of DA capacity and storage.	• Patient selection<br>• DA-rich graft survival and growth
11C-DASB	5-HT transporter (SERT)	Marker of presynaptic 5-HT terminals integrity and SERT availability	• Graft-derived 5-HT innervation and growth<br>• Graft-induced dyskinesias

11C-PE2I	Dopamine transporter (DAT)	Marker of presynaptic DA terminals integrity and DAT availability	• DA-rich graft survival and growth<br>• Graft-induced dyskinesias

All scans will take place at Imonova at the Hammersmith Hospital Campus, Imperial College London, United Kingdom. All 40 patients enrolled will undergo a PET protocol consisting of 18F-DOPA, 11C-DASB and 11C-PE2I, at baseline (0m), and then at 18 and 36 months (Table 2).

For any patients who have completed the baseline PET more than 9 months before the fetal transplant, both transplant and control patients will be required to have an additional PET scan prior to transplant consisting of 18F-DOPA only. This is to determine safety of the procedure and eligibility of the transplant and to match patients and controls.

Table 1 *Summary of PET studies*

PET studies	**BASELINE**	**18 Months or Baseline**	**36 Months**
18F-DOPA	**X**	**X**	**X**
11C-DASB	**X**	**X**	**X**
11C-PE2I	**X**	**X**	**X**

•

Baseline Visit

After review of the PET scan and confirmation that the patient remains eligible for a transplant, each patient will be notified whether they have been selected at random for transplantation. An appointment will be made for the 20 patients selected for transplantation to attend Addenbrooke's hospital within 28

days of surgery. All data collected from local screening visits will be checked and the following assessments will be completed:

- Height
- Weight
- Anaesthesia assessment
- Concomitant medications
- Vital signs – Blood pressure, pulse
- Adverse Events
- ECG
- Decision regarding need and timing of pre-operative withdrawal of any PD medication.

Following the successful completion of the all the above assessments the patient will attend Addenbrooke's Hospital to be admitted for surgical therapy the day before planned surgery.

Day before Surgery

The patient will receive the following according to patient requirements:

- Immunosuppressant as stated in section 9.1.7

Day of Surgery

The following assessments must be completed on the day of surgery:

- Vital Signs
- FBC, CRP
- Urea, creatinine and electrolytes
- Liver Function
- AEs
- PD medication and concomitant medication

Patients will then proceed to theatre for neural grafting.

Follow Up Procedures for the first 12, 24 and 48 hours post surgery

Patients are required to complete the following assessments:

- Vital Signs
- FBC (24 and 48 hours only)
- Urea, creatinine and electrolytes, CRP (24 and 48 hours only)
- Cy A Level (24 and 48 hours only)
- Liver Function (24 and 48 hours only)

- AEs
- MRI Scan of the brain 48-72 hours post surgery using Susceptibility Weighted Sequences (SWI)
- PD medication and concomitant medication

When patients are discharged from hospital they will rejoin the observational study.

Follow up procedures 7, 14, 21, 28 and 42 days post surgery

- FBC
- Urea, creatinine and electrolytes
- CMV
- Cy A Level
- Liver Function
- AEs
- PD medication and concomitant medication

Follow up procedures 2, 3, 4, 5, 6, 9 and 12 months post surgery

- FBC
- Urea, creatinine and electrolytes
- CMV
- Cy A Level
- Liver Function
- AEs
- PD medication and concomitant medication

Other Study-related visits

Patients will re join the observational study post surgery and continue their usual visits and assessment. They will continue to have safety follow up on the transplant study, until patient withdrawal.

Patients will sign appropriate local hospital consent forms for procedures that they will undergo in this study. These will include imaging, surgery and a declaration of intent for the use of their brains for research purposes (optional).

Schedule of assessments

Assessment	**Screening/ Baseline**		**Hours**				**Days**					**Months**						
	**-90 days - Day -1**		**Day 0 Surgery**	**12**	**24**	**48**	**7**	**14**	**21**	**28**	**42**	**2**	**3**	**4**	**5**	**6**	**9**	**12**
Observational Study 12 month	X																	
Informed Consent	**X**																	
Entry Criteria		X																
PD Medication		X					X	X	X	X	X	X	X	X	X	X	X	X
Concomitant Medication		All Visits																
Height		X																
Weight		X																
Vital Signs		X	X	X	X	X												
Physical Examination	X																	X
FBC	X		X		X	X	X	X	X	X	X	X	X	X	X	X	X	X
U+E	X		X		X	X	X	X	X	X	X	X	X	X	X	X	X	X
Cy A Level					X	X	X	X	X	X	X	X	X	X	X	X	X	X
Liver Function	X		X		X	X	X	X	X	X	X	X	X	X	X	X	X	X
ECG		X																
MRI Scan			X			X												
PET Scan	X																	

Continue with Observational Study						X												
Microbiology / Serology	X																	
Immunosuppressant		To be taken as prescribed																
Adverse Event	All Visits																	

Assessment of Safety

For the purpose of this study, an Adverse Event (AE) is defined as any untoward medical occurrence or experience in a participant which occurs from the point of patient registration until 28 days after last study assessment in this transplant trial (in this case the last dose of immunosuppressive medication), regardless of the dose or causal relationship. This includes any unfavourable or unintended signs (such as rash or enlarged liver), or symptoms (such as nausea or chest pain), an abnormal laboratory finding (including blood tests, x-rays or scans) or a disease temporarily associated with the use of the protocol treatment.

An Adverse Reaction (AR) is defined as any response to a medical product, that is noxious, related to any dose.

A Serious Adverse Event (SAE) is defined as any adverse event occurring to a patient, whether or not considered related to the protocol treatment, which:

Leads to death;

Is life threatening;

Results in persistent or significant disability of incapacity;

Requires in-patient hospitalisation or prolongation of existing hospitalisation;

Consists of a congenital anomaly / birth defect.

The term "life-threatening" in the definition of "serious" refers to an event in which the patient was at risk of death at the time of the event; it does not refer to an event which hypothetically might have caused death if it were more severe.

An SAE which is considered related to the protocol treatment is defined as a Serious Adverse Reaction (SAR).

A Suspected Unexpected Serious Adverse Reaction (SUSAR) is any serious adverse reaction for which the nature or severity is not consistent with the applicable product.

The Investigator must assess the relationship of all SAEs to study therapy. The following definitions should be used:

Not Related - There is not a temporal relationship to the study therapy, or there is a reasonable causal relationship between another drug, concurrent disease or circumstance and the event.

Unlikely to be related - There is little evidence to suggest there is a causal relationship. There is another reasonable explanation for the event (e.g. the patient's clinical condition or concomitant treatments)

Possibly Related - The AE has a timely relationship to study therapy. However, a potential alternative aetiology exists, or dechallenge information is unclear.

Probably Related - The adverse event has a timely relationship to study therapy and a potential alternative aetiology is not apparent. The event responds to dechallenge.

Definitely Related - The AE has a timely relationship to study therapy and resolves when the drug is discontinued and a potential alternative aetiology is not apparent. Upon re-challenge with study therapy the event recurs.

'Unlikely' and 'Not Related' are considered not study therapy related.

'Definitely', 'Probably' and 'Possibly' are considered study therapy related.

Reporting procedure

Adverse Event Reporting and Treatment

All AEs occurring from registration until 28 days after the last study therapy administration, whether observed by the Investigator or reported by the patient during the study period, and whether or not they are considered related to the therapy, must be documented in the medical records and on the relevant CRF pages supplied.

If the AE resolves completely, or resolves to baseline, and then worsens again, this must be recorded as a separate AE event.

If more than one AE occurs, each event should be recorded separately.

The Investigator will take all therapeutic measures necessary for resolution of any AE. Any medication necessary for treatment of the adverse event must be recorded onto the concomitant medications section of the patient's eCRF.

Pre-existing conditions

A pre-existing condition must not be reported as an AE unless the condition worsens during the study. The condition, however, must be recorded in the appropriate section of the eCRF.

Diagnostic and Surgical Procedures

Diagnostic and therapeutic non-invasive and invasive procedures, such as surgery, must not be reported as an AE. However, the medical condition for which the procedure was performed must be reported as an AE, whilst the procedure must be reported under "Comments" on the AE page.

Follow-up of Adverse Events and Clinically Relevant Laboratory Abnormalities

All AEs that are not SAEs and are unrelated to the study therapy will be followed-up for 28 days after the last study therapy administration. If there are AEs that occurred while the patient was in the study which are attributed to the study therapy, the event will be monitored monthly until resolution, stabilisation of the event, or until it is diagnosed as a chronic condition. In the event that the patient commences further treatment, this must be recorded on the eCRF, and a final assessment of the AEs made for the purposes of this study.

Serious Adverse Event and SUSAR Reporting Procedure

Expected side effects –These AEs must be reported as SAEs if they fulfil any other SAE criteria.

Adverse Effects from Neurosurgery

1: Haemorrhage at site of grafting, clinically relevant

2: Stroke in peri-operative period

3: Infection at site of surgery

Adverse Effects from Transplantation

1: Misplacement of tissue into wrong location in brain

2: Cell overgrowth of implant causing mass effect

3: Immune response to transplant, causing local inflammation

Adverse Effects from Immunosuppressant

1: Renal and bone marrow impairment

2: Opportunistic infection

3: Long term risk of malignancy is slightly increased by some of the immunosuppressant's used.

Reporting of SAEs

Where a patient experiences more than one AE which fulfils the serious criteria, at the same time, the event term used for the report should be that which is the root / underlying / most

serious event. Associated signs, symptoms and events should be recorded as part of the narrative of the event.

In the event of a patient experiencing serious adverse events on different occasions, these experiences should be reported as separate events.

In the case of an SAE the Investigator or designee must immediately:

Complete a ‘Serious Adverse Event Form’.

The form should be completed and signed by an appropriate member of the site trial team and faxed (within 24 hrs of becoming aware of the event) to the BRC immediately on fax number: 01223 331174.

The Principal Investigator must counter sign as soon as possible.

Even if only limited information is initially available, this should be provided and faxed on an SAE form. Further details should be submitted as soon as they become available.

In the case of death, life-threatening events or SUSARs:

TELEPHONE (on day of awareness) the BRC, Tel: 01223 331160.

This is in addition to faxing an SAE form to the BRC.

Post-mortem data, where available, for deaths occurring from registration until 28 days after the study treatment should be provided to the BRC. In addition, available post-mortem data should be provided for any death occurring after this time, if the death is considered to be possibly related to the experimental therapy.

The BRC will inform the Research Ethics Committee (REC), any other regulatory authorities including TransEUro clinical trial monitoring committee where appropriate. All reportable events (serious and unexpected, and drug related/unknown relationship, and any others as advised by the relevant safety and ethics committees and authorities), will be sent to Investigators.

The BRC will send a safety report to Local Research Ethics Committee annually and a copy to all investigational sites.

Follow-up of SAEs

In the case of an SAE, the subject must be followed-up until clinical recovery is complete and laboratory results have returned to normal, or until the condition has stabilised / assessed as being a chronic condition.

All SAEs that are related to the study therapy and still present at the end of the study, must be followed at least until the final outcome is determined, even if it implies that the follow-up continues after the patients leave the trial, and when appropriate until the end of the planned period of follow-up.

The Investigator and others responsible for patient care should instigate any supplementary investigations of significant AEs based on their clinical judgement of the likely causative factors. This may include seeking a further opinion from a specialist in the field of the AE.

In agreeing to the provisions of this protocol, these responsibilities are accepted by the Investigator.

Statistics and sample size

Statistical Power

The trial will involve comparison of the change in UPDRS at 36 months post-grafting between the treatment and control group. The standard deviation for the change in the UPDRS score in the control group is approximately 0.6 (Evans et al, unpublished data). Assuming the same standard deviation in the treatment group, a sample size of 20 patients in the treatment group being compared to 130 patients in a control group would give 80% power to detect a mean outcome difference of 0.4 units on the UPDRS scale at the 95% significance level.

Statistical analysis

Statistical analysis will be undertaken by experienced statisticians, independent from the research team to ensure unbiased interpretation of the results. A plan for the analysis will be comissioned prior to the completion of the study to be executed after all 20 patients have been followed up for 36 months, the data has been collected and monitored for quality control reasons. We will include a comparison of the 20 transplanted patients, compared with 20 imaged patients, compared with the remaining patients in the observational study.

Study Management

Investigator authorisation procedure

This study will require approval from the relevant regulatory and health authorities that apply within that country. Approval must be obtained from the relevant Independent Ethics Committee (IEC) of the local hospital. The IEC will conform to the standards of ICH E6. IEC approval must be received in writing. All approvals must be in place prior to any patient recruitment. The Principal Investigator must provide the following core documentation and attend an initiation visit before the site becomes activated:

- Site contact details, include contact details for research nurse and PI;
- Completed delegation of responsibilities and signature log;
- Up-to-date, signed and dated, Curriculum Vitae (CV) for each individual on the signature and responsibilities log, including dates of ICH-Good Clinical Practice (ICH-GCP) training;
- Written confirmation of local hospital approval for the study;
- Copies of the patient information sheet, GP information letter and consent form on local headed paper, as they are to be used in the study.
- Further documentation may be required due to changes to the protocol or changes to local policy. These will be discussed with, and requested from the local investigator when required.

Patient registration procedure

Once a patient is confirmed as eligible for the study, and has given written informed consent, the Investigator or designee will complete the registration forms supplied in the eCRF.

Case Report Forms and procedures for collecting data

Site monitoring

At the site initiation visit the study monitor will review the protocol, eCRF and specific study procedure with the investigators and their staff.

During the study, the study monitor will regularly check the completeness of patient case records, the accuracy of entries on the eCRFs, the adherence to the protocol and to Good Clinical Practice (GCP) and the progress of enrolment. Key trial personnel must be available to assist the monitor during these visits.

The investigator must give the monitor access to all relevant source documents to confirm their consistency with the eCRF entries. The monitoring standard requires full

verification for the presence of informed consent, adherence to the inclusion/exclusion criteria, documentation of SAEs, and the recording of data that will be used for all primary and safety variables. No information in source documents about the identity of the subjects will be disclosed.

The investigator must maintain source documents for each patient in the study, consisting of case and visit notes (hospital or clinic medical records) containing demographic and medical information, laboratory data, electrocardiograms, and the results of any other tests or assessments. All information on eCRFs must be traceable to these source documents in the patient's file. Data not requiring a separate written record will be defined before study start and will be recorded directly on the eCRFs, which will be documented as being the source data. The investigator must also keep the original informed consent form signed by the patient (a signed copy is given to the patient).

Case Report Form Procedures

12.3.2.1 Data management

The study database of the Secutrial® system of the Central Information Office Marburg will be used. Secutrial® is an internet based system with connection to a rational ORACLE® data base.

The software serves as remote data entry system for pseudonymized medical data. It includes functions for data entry in electronic forms, for data view, analysis and export.

The software development was done strictly in accordance to a standardized procedural model, meeting all ISPE GAMP4 requirements of software validation.

SecuTrial® is permanently audited to meet all requirements according to GCP, AMG, EMEA and FDA (21 CFR Part 11). Last audit was in September 2010.

Data management based on GCP refers to the activities defined to achieve safe routines to efficiently enter patient information into a database, avoiding errors.

The data management routines include procedures for handling of CRF, database set-up and management, data entry and verification, data validation, quality control (QC) of database, and documentation of the performed activities including information of discrepancies in the process. The database, data entry screens, and program will be designed in accordance with the clinical study protocol.

12.3.2.2 The entering of data into the CRF

The CRFs should always reflect the latest observations on the patients participating in the study. Therefore, the CRFs are to be completed as soon as possible during or after the patient's visit. To avoid inter-observer variability, every effort should be made to ensure that the same individual who made the initial baseline determinations completes all effect and safety evaluations. The principal investigator must verify that all data entries in the CRFs are accurate and correct. If some assessments are not done, or if certain information is not available, not applicable or unknown, the principal investigator should indicate this in the CRF. The principal investigator will be required to electronically sign off adverse and serious adverse event reports in the study database.

12.3.2.3 Source Documents

The CRF is essentially considered a data entry form and should not constitute the original (or source) medical records unless otherwise specified. Source documents are all documents used by the principal investigator or hospital that relate to the patient's medical history, that verify the existence of the patient, the inclusion and exclusion criteria, and all records covering the patient's participation in the study. They include laboratory notes, memoranda, material dispensing records, patient files, etc.

The principal investigator is responsible for maintaining source documents. These will be made available for inspection by the study monitor at each monitoring visit. The principal investigator must submit a completed CRF for each patient who signed the Patient Informed Consent. All supportive documentation submitted with the CRF, such as laboratory or hospital records should be clearly identified with the study and Patient number. Any personal information, including patient name, should be removed or rendered illegible to preserve individual confidentiality.

12.3.2.4 Audit Trail

All changes will be fully recorded in a protected audit trail, and a reason for the change will be required. Once all data have been entered, verified, and validated, the database will be locked.

12.3.2.5 Patient Confidentiality

The patients have the right to request access to his/her personal data and the right to request rectification of any data that is not correct and/or complete. The PI or designee personnel whose responsibilities require access to personal data agree to keep the identity of the patients confidential. This agreement is to be substantiated in a separate document.

12.3.2.6 Audits and Inspections

The purpose of an audit or inspection is to systematically and independently examine all study-related activities to document that they were conducted, recorded, analysed and accurately reported according to the study protocol and the background regulatory demands.

Audits or inspections may therefore be performed at the study site during or after the study. Visits may thereby be paid by the authorized monitors for the study (this may be a company), by a regulatory authority or an ethics committee. These visits may include source data verification and confidentiality documents are therefore created.

The investigator should contact the appointed monitors immediately if they are contacted by a regulatory agency about an inspection at their study site.

12.3.2.7 Training of Study Personnel

The principal investigator will maintain records of all individuals involved in the study (medical, nursing, pharmaceutical and other staff). The principal investigator will ensure that appropriate training relevant to the study is given to the staff involved in the study, and that any new information of relevance to the conduct of the study is forwarded to the persons involved.

12.3.2.8 Study Reporting

A Clinical Study Report, according to the ICH Guideline for Structure and Content of Clinical Study Reports, will be prepared by the monitor in close collaboration with the Investigator, the trial statistician and the data management team. All publications and presentations must be based upon the Study Report. Thus, no party will communicate any result of the study to a third party before the study report has been prepared.

12.3.2.9 Database quality control

Data from the CRFs are entered into the study database by the clinical investigator’s staff, using single data entry with electronic verification.

Subsequently, the entered data are systematically checked by the monitor, using the internal query system of the medical database, completeness reports and printable queries details lists. The medical database system contains several completeness and plausibility data checks. Case report forms can only be stored after answer completeness and plausibility errors with correct data entries. Quality control audits of all key safety and efficacy data in the database are made prior to locking the database.

Concomitant medications entered into the database will be coded using the WHO Drug Reference List, which employs the Anatomical Therapeutic Chemical classification system. The medical database offers the official ATC-Code catalogue for adequate ATC coding. Medical history/current medical conditions and adverse events will be coded by the PI using the Medical dictionary for regulatory activities (MedDRA) terminology.

At the conclusion of the study, the occurrence of any protocol violations will be determined. After these actions have been completed and the database has been declared to be complete and accurate, it will be locked and made available for data analysis.

Essential Documentation and Archiving

The Investigator will be provided with an Investigator Site File prior to opening to recruitment. This file contains all essential documentation pertaining to the study, and must be safely archived by the Investigator, after completion of the study.

All essential source and study documentation must be securely retained by participating centres for at least 15 years, or the length of time specified by current, applicable legislation, whichever is the longer, after the Sponsor has confirmed that the study has ended.

The Investigator must not destroy any documents or records associated with the study without prior approval from the Sponsor.

Amendments

Amendments to, or formal clarifications of, the study protocol will be documented in writing. BRC will remain responsible for all amendments or administrative changes to the protocol,

and will distribute up-to-date documentation upon appropriate regulatory body approval to each specific site for their own regulatory approval.

All Investigators will acknowledge the receipt of new study documentation, and remain responsible for gaining local Trust approval before implementing any changes. Written confirmation of local trust approval must be sent to the BRC.

Any amendment affecting the patient requires the patient's informed consent before implementation.

Trial sponsorship and financing

This study is a clinician-initiated and clinician-led study funded from European Commission Seventh Framework Programme funds. The study is being run by Addenbrooke's Hospital. Cambridge University Hospitals National Health Service (NHS) Foundation Trust and the University of Cambridge is the joint study Sponsor:

R&D Manager, R&D Department (Box 277),

Addenbrooke's Hospital, Hills Road, Cambridge, CB2 0QQ,

Tel No: 01223 245151

Email: r&denquiries@addenbrookes.nhs.uk

Trial insurance

The University Insurance Manager has advised that insurance for negligent and non negligent harm under the University's Clinical Trial policy can be arranged if this trial is approved by the Local Research Ethics Commitee. The University's insurers are Newline, the insurance policy reference is B0823Q31000177 and the Limit of Indemnity under the policy is £10m.

Ethical Considerations

Patient protection

This study will be carried out in accordance with the World Medical Association (WMA) Declaration of Helsinki (2008). Copies of the declaration may be obtained by contacting the BRC, or from WMA website: http://www.wma.net/e/policy/b3.htm. Prior approval must be obtained by the relevant IEC of the local hospital. The IEC will be provided with all study documentation and any amendments. All approvals must be received in writing.

The protocol has been written, and the study will be conducted according to the EU Directive 2001/20/EC, EU Directive 2005/28/EC.

Prior to any potential patient being approached, all regulatory approvals will be acquired.

Informed consent

All patients will be informed of the aims of the study, the treatment and possible hazards to which they will be exposed. The patient will be informed as to the strict confidentiality of their patient data, but that their medical records may be reviewed for trial purposes by authorised individuals other than the treating physician. Informed consent documents will be provided by the Sponsor on Trust headed letter paper.

It will be emphasised that the participation is voluntary and that the patient is within their rights to withdraw from the study without giving reason. This will not prejudice the patient's subsequent care. Documented informed consent must be obtained for all patients included in the study before they are registered in the study. This must be done in accordance with the national and local regulatory requirements.

For European Union member states, the informed consent procedure must conform to the ICH-GCP guidelines. This implies that "the written informed consent form should be signed and personally dated by the patient or by the patient's legally acceptable representative".

In the event of consent documentation requiring translation

It is the responsibility of the individual Investigator to translate the enclosed informed consent document. The translated version must be dated and version controlled and sent to the Sponsor prior to use. It is the responsibility of the Investigator to ensure that the translation is conforming to the ICH-GCP guidelines.

All sections of the informed consent document must appear in the translation.

Publication Policy and Press Releases

The main trial results will be published in the name of the trial in a peer reviewed journal, on behalf of all collaborators. The manuscript will be prepared by the study team. The BRC and all participating centres and Investigators will be acknowledged in this publication. All presentations and publications relating to the trial must be authorised by the study team.

References

1. Braak H, Ghebremedhin E, Rub U et al. Stages in the development of Parkinson's disease related pathology, Cell Tissue Res 2004 318: 121-134.
2. Braak H, Bohl JR, Muller CM et al. Stanley Fahn Lecture 2005: The staging procedure for the inclusion body pathology associated with sporadic Parkinson's disease reconsidered, Mov Disord 2006 21: 2042-2051.
3. Lang AE and Lozano A. Parkinson's disease. New Engl.J.Med 1998 338: 113-1143 and 1144-1153.
4. Lewis SJ, Caldwell MA, Barker RA. Modern theraputic approaches in Parkinson's disease. Expert Rev Mol Med. 2003: 1-20.
5. Witt K, Daniels C, Reiff J, Krack P, Volkmann J, Pinsker MO, Krause M, Tronnier V, Kloss M, Schnitzler A, Wojtecki L, Bottzel K, Danek A, Heilker R, Strum V, Kupsch A, Karner E, Deuschl G. Neuropsychological and psychiatric changes after deep brain stimulation for Parkinson's disease: a randomised, multicentre study. Lancet Neurol. 2008 7: 605-14.
6. Voon V, Krack P, Lang AE, Lozano AM, Dujardin K, Schupbach M, D'Ambrosia J, Thobois S, Tamma F, Herzog J, Speelman JD, Samanta J, Kubu C, Rossignol H, Poon YY, Saint Cyr JA, Ardouin C, Moro E. A mulitcentre study on suicide outcome following subthalamic stimulation for Parkinson's disease . Brain. 2008 131: 2720-8.
7. Wider C, Pollo C, Bloch J, Burkhard PR, Vingerhoets FJ. Long term outcomes of 50 consecuative Parkinson's disease patients treated with subthalamic deep brain stimulation. Parkinsonism Relat Disord. 2008; 14: 114-9.
8. Evans JR, Barker RA. Neurotrophic factors as a theraputic target for Parkinson's disease. Expert Opin Ther Targets. 2008 12: 437-47.
9. Wijeyekoon R, Barker RA. Cell replacement therapy for Parkinson's disease. Biochim Biophys Acta. 2008 Epub. Oct 25.

10. Defer, GL, et al. Long term outcome of unilaterally transplanted parkinsonian patients. I. Clinical approach. Brain 1996 119:41-50
11. Freed, C.R., et al. Survival of implanted fetal dopamine cells and neurologic improvement 12 to 46 months after transplantation for Parkinson's disease. N Engl J Med, 1992. 327: 1549-55
12. Hagell, P. Et al, Sequential bilateral transplantation in Parkinson's disease: effects of the second graft. Brain, 1999. 122: 1121-1132.
13. Hauser, R.A., et al. Long term evaluation of bilateral fetal nigral transplantation in Parkinson's disease. Arch. Neurol. 1999. 56: 179-187.
14. Brundin, P. Et al. Bilateral caudate and putamen grafts of embryonic mesencephalic tissue treated with lazaroids in Parkinson's disease. Brain, 2000. 123: 1380-1390.
15. Mendez, I. Et al. Simultaneous intrastriatal and intranigral fetal dopaminergic grafts in patients with Parkinson's disease: a pilot study. Report of three cases. J Neurosurg 96: 589-596, 2002.
16. Freed, C.R. et al. Transplantation of embryonic dopamine neurons for severe Parkinson's disease. New Engl.J.Med. 2001. 344: 710-719.
17. Olanow, C.W. et al. A double blind controlled trial of bilateral fetal nigral transplantation in Parkinson's disease. Ann.Neurol. 2003. 54: 403-414.
18. Hagell, P, et al. Dyskinesias following neural transplantation in Parkinson's disease. Nat Neurosci. 2002; 5: 627-8
19. Piccini, P.et al. Dopamine release from nigral transplants visualised in vivo in a Parkinson's patient. Nature Neuroscience. 1999; 2, 1137-1140.

Appendix 1:

TRANSEURO PET PROTOCOL

A. Background

In previous clinical trials, positron emission tomography (PET) brain imaging has provided objective in vivo evidence that human dopamine (DA) -rich fetal ventral mesencephalic (VM) tissue implanted in the striatum of Parkinson's disease (PD) patients can survive, grow, release DA, normalize brain metabolism and restore striatal-cortical connections, clinically corresponding to significant symptomatic relief in some cases (Lindvall and Hagell, 2000; Brooks, 2004; Lindvall and Bjorklund, 2004).

Retrospective analysis of post-transplantation data has also allowed us to understand a number of ways that PET imaging could help in understanding and monitoring outcomes of fetal cell transplantation trials in PD. We have now up to 16 years of post-transplantation 18F-DOPA PET follow-up data showing graft viability and continuous clinical benefit in several transplanted PD patients with fetal VM tissue (Piccini et al, 1999; Ma et al., 2010; Politis et al., 2010a; 2011).

The degree of motor impairment has been shown to correlate with 18F-DOPA uptake in the striata of PD patients [Morish et al., 1996] and clinical outcomes following fetal cell transplantation can be associated with the number of viable DA cells innervating the striatum as assessed by 18F-DOPA PET. This correlation could explain the differences in the outcomes between open-label trials (reporting an average of 50 to 85% increase in 18F-DOPA uptake associated with good clinical improvement of PD motor symptoms and reductions in medication requirements (Freeman et al., 1995; Remy et al., 1995; Wenning et al., 1997; Hagell et al., 1999; Brundin et al., 2000)), and the two double-blind sham-surgery controlled clinical trials (reporting an average of 20 to 40% increases in 18F-DOPA uptake

associated with poor clinical outcomes during the first periods post-transplantation [Freed et al., 2001; Olanow et al., 2003]).

18F-DOPA PET can also help facilitate patient selection and screening as patients with baseline reductions in 18F-DOPA uptake extending to the ventral part of the striatum and patients with reductions in 18F-DOPA uptake consistent with atypical or secondary Parkinsonism should be excluded from these trials (Piccini et al., 2005; Ma et al., 2010). This knowledge, which derived from post-hoc analysis, raises the possibility of a time window for optimal transplantation outcomes as preoperative preservation of DA innervation in ventral striatal areas seems to be predictive of a better outcome.

Graft-induced dyskinesias (GIDs) are a significant adverse effect of fetal cell transplantation in PD tht has impacted on the further development of cell transplantation trials (Freed et al., 2001; Hagell et al., 2002; Ma et al., 2002; Olanow et al., 2003; 2009; Politis et al., 2010a; 2010b; 2011). Notwithstanding the several theories proposed (Politis, 2010b), recent data imply that the development of GIDs could be related to the composition of grafted tissue (Politis et al., 2010a), as human fetal VM tissue contains a varied proportion of non-DA neurons (Isacson et al., 2003), including serotonin (5-HT) neurons. 11C-DASB PET and a therapeutic challenge with an agent dampening transmitter release from 5-HT neurons was used to show a causal relationship between mishandling of DA release from the graft-derived 5-HT hyperinnervation in the striatum of PD patients and the occurrence of GIDs (Politis et al., 2010a; 2011). Moreover, it was suggested that the high ratio of 5-HT and DA neurons (and their respective transporters – SERT/DAT) could be the driving factor for the development of GIDs (Politis et al., 2010a; 2010b; 2011). Therefore, these results suggest that achieving normal striatal 5-HT/DA and SERT/DAT ratios following transplantation of fetal tissue would be desirable in avoiding the development of GIDs.

B. Aims of PET protocol assessing the outcome of fetal cell transplantation in

Parkinson's disease

The optimal functional imaging protocol using PET imaging in TRANSEURO (Table 1) will use two radioligands that specifically tag the DA presynaptic terminals in order to assess DA-rich graft survival and growth (18F-DOPA for AADC and 11C-PE2I for DAT). According to data from open-label and double blind trials it appears that a short-term increase (~18 months) in 18F-DOPA uptake of more than 50% from baseline is necessary to achieve clinically valuable antiparkinsonian effects. Measures of 18F-DOPA uptake at baseline will also provide valuable information to assist in the selection of patients for trials of cell-based DA therapies in PD by excluding those showing reduced uptake in the ventral striatum.

TRANSEURO PET imaging will also use a third radioligand that specifically tags the 5-HT presynaptic terminals (11C-DASB for SERT). 11C-DASB will be used to assess the graft-derived 5-HT innervation and growth in PD patients receiving intrastriatal transplants. 11C-DASB PET together with 18F-DOPA and 11C-PE2I PET will be used to calculate binding ratios reflecting the proportions of 5-HT to DA neurons and SERT to DAT binding sites.

Previous knowledge has shown that in order to efficiently monitor cell replacement therapies in PD with functional imaging, long follow- up assessments are needed and conclusions cannot be definitively drawn with short follow-up periods. Therefore, we will be assess outcomes with PET imaging pre-operationally and at 18 and 36 months following transplantation,

C. PET ligands

Table 2 *TRANSEURO PET ligands*

E. References

Brooks DJ. Positron emission tomography imaging of transplant function. NeuroRx. 2004 Oct;1(4):482-91.

Brundin P, Pogarell O, Hagell P, Piccini P, Widner H, Schrag A, Kupsch A, Crabb L, Odin P, Gustavii B, Björklund A, Brooks DJ, Marsden CD, Oertel WH, Quinn NP, Rehncrona S, Lindvall O. Bilateral caudate and putamen grafts of embryonic mesencephalic tissue treated with lazaroids in Parkinson's disease. Brain. 2000 Jul;123 (Pt 7):1380-90.

Freed CR, Greene PE, Breeze RE, Tsai WY, DuMouchel W, Kao R, Dillon S, Winfield H, Culver S, Trojanowski JQ, Eidelberg D, Fahn S. Transplantation of embryonic dopamine neurons for severe Parkinson's disease. N Engl J Med. 2001 Mar 8;344(10):710-9.

Freeman TB, Olanow CW, Hauser RA, Nauert GM, Smith DA, Borlongan CV, Sanberg PR, Holt DA, Kordower JH, Vingerhoets FJ, et al. Bilateral fetal nigral transplantation into the postcommissural putamen in Parkinson's disease. Ann Neurol. 1995 Sep;38(3):379-88.

Hagell P, Schrag A, Piccini P, Jahanshahi M, Brown R, Rehncrona S, Widner H, Brundin P, Rothwell JC, Odin P, Wenning GK, Morrish P, Gustavii B, Björklund A, Brooks DJ, Marsden CD, Quinn NP, Lindvall O. Sequential bilateral transplantation in Parkinson's disease: effects of the second graft. Brain. 1999 Jun;122 (Pt 6):1121-32.

Hagell P, Piccini P, Björklund A, Brundin P, Rehncrona S, Widner H, Crabb L, Pavese N, Oertel WH, Quinn N, Brooks DJ, Lindvall O. Dyskinesias following neural transplantation in Parkinson's disease. Nat Neurosci. 2002 Jul;5(7):627-8.

Isacson O, Bjorklund LM, Schumacher JM. Toward full restoration of synaptic and terminal function of the dopaminergic system in Parkinson's disease by stem cells. Ann Neurol. 2003;53 Suppl 3:S135-46; discussion S146-8.

Lindvall O, Hagell P. Clinical observations after neural transplantation in Parkinson's disease. Prog Brain Res. 2000;127:299-320.

Lindvall O, Björklund A. Cell therapy in Parkinson's disease. NeuroRx. 2004 Oct;1(4):382-93.

Ma Y, Feigin A, Dhawan V, Fukuda M, Shi Q, Greene P, Breeze R, Fahn S, Freed C, Eidelberg D. Dyskinesia after fetal cell transplantation for parkinsonism: a PET study. Ann Neurol. 2002 Nov;52(5):628-34.

Ma Y, Tang C, Chaly T, Greene P, Breeze R, Fahn S, Freed C, Dhawan V, Eidelberg D. Dopamine cell implantation in Parkinson's disease: long-term clinical and (18)F-FDOPA PET outcomes. J Nucl Med. 2010 Jan;51(1):7-15.

Morrish PK, Sawle GV, Brooks DJ. An [18F]dopa-PET and clinical study of the rate of progression in Parkinson's disease. Brain. 1996 Apr;119 (Pt 2):585-91.

Olanow CW, Goetz CG, Kordower JH, Stoessl AJ, Sossi V, Brin MF, Shannon KM, Nauert GM, Perl DP, Godbold J, Freeman TB. A double-blind controlled trial of bilateral fetal nigral transplantation in Parkinson's disease. Ann Neurol. 2003 Sep;54(3):403-14.

Olanow CW, Gracies JM, Goetz CG, Stoessl AJ, Freeman T, Kordower JH, Godbold J, Obeso JA. Clinical pattern and risk factors for dyskinesias following fetal nigral transplantation in Parkinson's disease: a double blind video-based analysis. Mov Disord. 2009 Feb 15;24(3):336-43.

Piccini P, Brooks DJ, Björklund A, Gunn RN, Grasby PM, Rimoldi O, Brundin P, Hagell P, Rehncrona S, Widner H, Lindvall O. Dopamine release from nigral transplants visualized in vivo in a Parkinson's patient. Nat Neurosci. 1999 Dec;2(12):1137-40.

Piccini P, Pavese N, Hagell P, Reimer J, Björklund A, Oertel WH, Quinn NP, Brooks DJ, Lindvall O. Factors affecting the clinical outcome after neural transplantation in Parkinson's disease. Brain. 2005 Dec;128(Pt 12):2977-86.

Politis M, Wu K, Loane C, Quinn NP, Brooks DJ, Rehncrona S, Bjorklund A, Lindvall O, Piccini P. Serotonergic neurons mediate dyskinesia side effects in Parkinson's patients with neural transplants. Sci Transl Med. 2010a Jun 30;2(38):38ra46.

Politis M. Dyskinesias after neural transplantation in Parkinson's disease: what do we know and what is next? BMC Med. 2010b Dec 2;8:80.

Politis M. Optimizing functional imaging protocols for assessing the outcome of fetal cell transplantation in Parkinson's disease. BMC Med. 2011 May 10;9:50.

Politis M, Oertel WH, Wu K, Quinn NP, Pogarell O, Brooks DJ, Bjorklund A, Lindvall O, Piccini P. Graft-induced dyskinesias in Parkinson's disease: High striatal serotonin/dopamine transporter ratio. Mov Disord. 2011 May 24. doi: 10.1002/mds.23743.

Remy P, Samson Y, Hantraye P, Fontaine A, Defer G, Mangin JF, Fénelon G, Gény C, Ricolfi F, Frouin V, et al. Clinical correlates of [18F]fluorodopa uptake in five grafted parkinsonian patients. Ann Neurol. 1995 Oct;38(4):580-8.

Wenning GK, Odin P, Morrish P, Rehncrona S, Widner H, Brundin P, Rothwell JC, Brown R, Gustavii B, Hagell P, Jahanshahi M, Sawle G, Björklund A, Brooks DJ, Marsden CD, Quinn NP, Lindvall O. Short- and long-term survival and function of unilateral intrastriatal dopaminergic grafts in Parkinson's disease. Ann Neurol. 1997 Jul;42(1):95-107.

Appendix 2:

TRANSEURO

TRANSEURO is a European research consortium with the principal objective to develop an efficacious and safe treatment methodology for **Parkinson’s disease suffering patients** using fetal cell based treatments. The consortium has gathered international experts including leading clinicians, scientists, industrial partners,

ethicists and patients' representatives who have joined forces in a new round of experimental work and cell therapy trials in Parkinson's Disease.

TRANSEURO is coordinated by **Prof. Roger Barker** from the University of Cambridge and is funded by a 7th Framework Program from the European Commission (Contract number HEALTH-F5-2010-242003).

Principal goals of TRANSEURO are:

- To show that the consistency and efficacy of dopaminergic cell replacement in Parkinson's disease can be improved by careful attention to tissue preparation and delivery, patient selection and immunosuppressive treatment
- To show that dopaminergic cell replacement can be clinically efficacious in the absence of any troublesome off-state dyskinesias in clinical trials of fetal ventral mesencephalic transplants in patients with mild Parkinson's disease
- To develop a protocol that can serve as a template for all future clinical trials in the cell therapy field including stem cell-based therapies and the ethical implications and ramifications of such work.

To achieve these aims the TRANSEURO partners aim to engage in a series of investigator lead, collaborative clinical studies which will be conducted in parallel across all of the EU partners (in France, Sweden and Germany).

TRANSEURO clinical projects:

- Observational study
    - Longitudinal follow-up of Parkinson's disease patients who are believed to be representative of the group of patients most likely to benefit from cell replacement therapies.
    - 300 patients recruited Europe wide
    - UK sites: Cambridge, University College London, Imperial College London & University Hospital, Wales (all sponsored by University of Cambridge and regulated by ethical approval 10/0304H/77)
    - European sites: Freiburg, Germany; Lund, Sweden & Paris, France (each country is responsible for its own sponsorship and regulatory approvals).

- Open label study
    - To assess the safety and efficacy of dopaminergic cell replacement therapies as a treatment for Parkinson's disease.
    - 40 patients to be recruited Europe wide (20 will be randomly selected for transplantation and 20 will be matched controls who will receive the same scanning protocol but WILL NOT receive any surgical procedures)
    - Patients recruited from the observational study only.
    - Remaining patients not selected for transplantation/matched controls will be used as a natural history control arm.
    - Observational protocol will be used to assess efficacy of transplants.

- UK sites: Cambridge (sponsored by CUHNFT and regulated by National Research Ethics Service)
- EU sites: Freiburg & Lund (each country is responsible for its own sponsorship and regulatory approvals).

THIS ETHICAL APPLICATION ONLY RELATES TO THE UK INVOLVEMENT IN THE TRANSPLANT STUDY. ALL OTHER EU COUNTRIES ARE RESPONSIBLE FOR THEIR OWN PROTOCOL AND REGULATORY SUBMISSIONS.
