# Supplementary material for: The TransEuro open-label trial of human fetal ventral mesencephalic transplantation in patients with moderate Parkinson’s disease
Source: Nat Biotechnol. 2025 May 2;44(1):70–8. doi: 10.1038/s41587-025-02567-2 (PMC12807853; doi:10.1038/s41587-025-02567-2)
Supplement: Supplementary file 2 — Reporting Summary [file 41587_2025_2567_MOESM2_ESM.pdf]

nature portfolio

Corresponding author(s): Roger A Barker; NBT-RA60604
Last updated by author(s): 19/12/24

Reporting Summary

Nature Portfolio wishes to improve the reproducibility of the work that we publish. This form provides structure for consistency and transparency in reporting. For further information on Nature Portfolio policies, see our Editorial Policies and the Editorial Policy Checklist.

Statistics

For all statistical analyses, confirm that the following items are present in the figure legend, table legend, main text, or Methods section.

n/a	Confirmed	
☐	☒	The exact sample size (*n*) for each experimental group/condition, given as a discrete number and unit of measurement
☐	☒	A statement on whether measurements were taken from distinct samples or whether the same sample was measured repeatedly
☐	☒	The statistical test(s) used AND whether they are one- or two-sided<br>*Only common tests should be described solely by name; describe more complex techniques in the Methods section.*
☐	☒	A description of all covariates tested
☐	☒	A description of any assumptions or corrections, such as tests of normality and adjustment for multiple comparisons
☐	☒	A full description of the statistical parameters including central tendency (e.g. means) or other basic estimates (e.g. regression coefficient) AND variation (e.g. standard deviation) or associated estimates of uncertainty (e.g. confidence intervals)
☐	☒	For null hypothesis testing, the test statistic (e.g. *F*, *t*, *r*) with confidence intervals, effect sizes, degrees of freedom and *P* value noted<br>*Give P values as exact values whenever suitable.*
☒	☐	For Bayesian analysis, information on the choice of priors and Markov chain Monte Carlo settings
☒	☐	For hierarchical and complex designs, identification of the appropriate level for tests and full reporting of outcomes
☐	X	Estimates of effect sizes (e.g. Cohen's *d*, Pearson's *r*), indicating how they were calculated

Our web collection on statistics for biologists contains articles on many of the points above.

Software and code

Policy information about availability of computer code

Data collection	No software was used
Data analysis	Standard approaches were used for PET image processing and kinetic modelling and for statistical analysis, utilising already published software cited in-text. R (version 4.3.1).MIAKAT™ v4.3.13 (Molecular Imaging and Kinetic Analysis Toolbox) [24] [25] implemented within MATLAB® 2016b (Mathworks, Natick, MA, USA), SPM12v7487 (Statistical Parametric Mapping, Wellcome Trust Centre for Neuroimaging, London, UK) and FSL v6 0 (FMRIB Image Analysis Group, Oxford, UK) (Jenkinson)

For manuscripts utilizing custom algorithms or software that are central to the research but not yet described in published literature, software must be made available to editors and reviewers. We strongly encourage code deposition in a community repository (e.g. GitHub). See the Nature Portfolio guidelines for submitting code & software for further information.

Data

Policy information about availability of data

All manuscripts must include a data availability statement. This statement should provide the following information, where applicable:

- Accession codes, unique identifiers, or web links for publicly available datasets
- A description of any restrictions on data availability
- For clinical datasets or third party data, please ensure that the statement adheres to our policy

The individual de-identified participant data (including data dictionaries) can be shared upon request.;
We can share the basic clinical data on all the patients;

We are happy to share the study protocol and the eventual statistical analysis plan as the trial changed from that which was planned originally; Data may be requested from the corresponding author upon reasonable request immediately and for a period of 36 months following article publication. Reasonable requests will be considered from researchers who provide a methodologically sound proposal. Data would be provided anonymised.

Human research participants

Policy information about studies involving human research participants and Sex and Gender in Research.

Reporting on sex and gender	The gender of the cohort is specified and was not specifically designed around this variable as we simply chose patients at random from a cohort and then they had to decide whether they would take part in the trial.
Population characteristics	Patient demographics are provide in Table 1 in the main manuscript. Age range of patients recruited was between 43-56.
Recruitment	Patients were recruited from TransEuro study. At the beginning of the transplant study, the patients were checked against the inclusion/exclusion criteria.
Ethics oversight	Informed consent was part of the ethical approval which is in the clinical trial protocol. Ethics committee Cambridgeshire Central Research Ethics Committee. The ethics approval statement is in the approval letter in the clinical trial protocol.

Note that full information on the approval of the study protocol must also be provided in the manuscript.

Field-specific reporting

Please select the one below that is the best fit for your research. If you are not sure, read the appropriate sections before making your selection.

☒ Life sciences ☐ Behavioural & social sciences ☐ Ecological, evolutionary & environmental sciences

For a reference copy of the document with all sections, see nature.com/documents/nr-reporting-summary-flat.pdf

Life sciences study design

All studies must disclose on these points even when the disclosure is negative.

Sample size	Final sample size was not obtained as explained in the manuscript due to issues of tissue availability.
Data exclusions	None.
Replication	This was a trial and so replication was not possible
Randomization	36 patients were randomised to join the transplant arm of the study, but 9 withdrew during pre-randomisation assessment.
Blinding	There was no blinding of the group in the traditional sense, but patients were assessed by a blinded third party using video recordings

Reporting for specific materials, systems and methods

We require information from authors about some types of materials, experimental systems and methods used in many studies. Here, indicate whether each material, system or method listed is relevant to your study. If you are not sure if a list item applies to your research, read the appropriate section before selecting a response.

Materials & experimental systems

n/a	Involved in the study	
☒	☐	Antibodies
☒	☐	Eukaryotic cell lines
☒	☐	Palaeontology and archaeology
☒	☐	Animals and other organisms
☐	☒	Clinical data
☒	☐	Dual use research of concern

Methods

n/a	Involved in the study	
☒	☐	ChIP-seq
☒	☐	Flow cytometry
☐	☒	MRI-based neuroimaging

Clinical data

Policy information about clinical studies

All manuscripts should comply with the ICMJE guidelines for publication of clinical research and a completed CONSORT checklist must be included with all submissions.

Clinical trial registration	NCT01898390

Study protocol	It is in the manuscript and also discussed in Barker RA et al Nature Medicine 2019
Data collection	The data was collected from 2011-2021 and is stored on a database at the University of Cambridge
Outcomes	Primary and secondary outcomes are described in the trial protocol which is the supplementary document.

Magnetic resonance imaging

Experimental design

Design type	Structural scans used only to aid the processing of PET images and to define regions of interest and also post-surgery to check transplant location and any haemorrhage or other post operative complication
Design specifications	n/a
Behavioral performance measures	n/a

Acquisition

Imaging type(s)	Structural
Field strength	3T
Sequence & imaging parameters	T1 MPRAGE (TR/TE = 2300/2.98ms; flip angle = 9; TI = 900ms; GRAPPA factor = 2; field of view = 240*256mm; matrix size = 240*256)
Area of acquisition	Whole brain

Diffusion MRI ☐ Used ☒ Not used

Preprocessing

Preprocessing software	MIAKAT v4.3.13, SPM12 v7487, FSL v6.0
Normalization	Normalisation not included as part of pipeline due to invasive nature of therapy. Data were instead extracted from ROIs in native space and statistics employed on these data outside of the images.
Normalization template	Not normalized
Noise and artifact removal	N/A
Volume censoring	N/A

Statistical modeling & inference

Model type and settings	N/A
Effect(s) tested	N/A

Specify type of analysis: ☐ Whole brain ☒ ROI-based ☐ Both

Anatomical location(s): Regions were identified using anatomical landmarks as described in Tziortzi et al. (2011) doi: 10.1016/j.neuroimage.2010.06.044

Statistic type for inference (See Eklund et al. 2016)	N/A
Correction	N/A

Models & analysis

n/a	Involved in the study
☒	☐ Functional and/or effective connectivity
☒	☐ Graph analysis
☒	☐ Multivariate modeling or predictive analysis
